# Multi‐omics analysis revealed biomarkers for coronary atherosclerosis: Occurrence and development

**DOI:** 10.1002/ctm2.70451

**Published:** 2025-08-21

**Authors:** Mengxue He, Dongxue Wang, Yong‐Jiang Xu, Jiachen Shi, Aiyang Liu, Xiaoxi Zhao, Yunlai Gao, Yuan He, Yu Zhang, Ru‐Xing Wang, Yuanfa Liu

**Affiliations:** ^1^ State Key Laboratory of Food Science and Technology School of Food Science and Technology National Engineering Research Center for Functional Food National Engineering Laboratory for Cereal Fermentation Technology, Collaborative Innovation Center of Food Safety and Quality Control in Jiangsu Province, Jiangnan University Wuxi China; ^2^ Department of Cardiology Wuxi People's Hospital Affiliated to Nanjing Medical University Wuxi China

**Keywords:** biomarker panel, coronary atherosclerosis, diagnostic, plasma metabolites

## Abstract

**Background:**

Coronary atherosclerosis (CA) is a leading cause of cardiovascular diseases with the high morbidity and mortality; however, the current diagnostic methods, primarily based on symptoms, signs, lab examination and imaging, are often inadequate for detecting subclinical or early‐stage CA, costly, and inaccessible in many cases. The objective of this study was to discover sensitive and specific biomarkers for the diagnosis of CA severity.

**Methods:**

We enrolled 443 participants, including CA patients and healthy controls, from three independent cohorts: discovery, testing, and blinded validation. Multi‐omics data integration during the discovery phase identified key features of atherosclerotic progression and potential biomarkers. Biomarker panels were refined using random forest models in the testing cohort, and their performance was evaluated in a blinded validation cohort to assess their ability to monitor the occurrence and development of CA.

**Results:**

Multi‐omics analysis revealed that plasma metabolites exhibited the strongest correlation with CA severity, effectively distinguished different CA stages from healthy controls. *Post hoc* analysis confirmed the diagnostic model's robustness, with an AUC value higher than .933 (95% CI: .828–.984, sensitivity 93.75%, and specificity 80%). In the blinded validation cohort, the biomarker panel achieved AUC values of .821–.898 for CA occurrence and .649–.849 for CA severity. Notably, 90% of these biomarkers remained significant after adjusting for comorbidities (*p* < .05).

**Conclusions:**

This study identified significant metabolic changes during CA progression and established biomarker panels with potential diagnostic value for assessing CA severity. Key metabolites including cholesteryl sulphate, azelaic acid, tryptophan, arabinofuranosyluracil, TMAO, ADMA, LPC18:2, tartaric acid, *L*‐citrulline, and *L*‐proline, purine, sorbitol, and 2‐aminoadipic acid. These findings highlight the potential of these biomarkers to improve early diagnosis and personalised management of CA.

## Graphical abstract

This finding could lead to clinical diagnosis of CA severity and provide new treatment strategies. A novel panel composed of five metabolites can be used to accurately diagnose the development of CA.

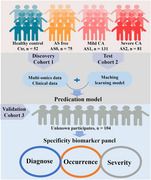

## 1 INTRODUCTION

Cardiovascular diseases (CVDs) have emerged as the leading cause of global mortality and morbidity, resulting in 17.9 million deaths annually.^1^ Atherosclerosis (AS) is recognised as the main pathological manifestation of most CVDs, affecting the entire vasculature as well as the coronary arteries. The intricate etiology and systemic impacts of AS, coupled with its severity, have contributed to its status as a highly studied disease.^2^ Coronary atherosclerosis (CA) is a primary CVD and serves as an indicator for coronary artery disease (CAD).^3^

Currently, coronary artery angiography (CAG) is still considered the gold standard for diagnosing CA. However, this procedure is invasive and associated with significant costs to both individual patients and the healthcare system due to the accompanying procedural risks. CAG is typically employed when clinical and biochemical factors indicate the presence of CA, and its preventative utility is limited. Notably, only 38% of patients without known heart diseases who underwent elective invasive angiography were found to have obstructive CAD.^4^ Biomarkers are helpful for the diagnosis and enhance our understanding of disease mechanisms, potentially leading to improved clinical decision‐making for prevention and treatment. Therefore, it is urgent to discover novel biomarkers for assessing the occurrence and development of CA.

Numerous studies have shown that omics analysis is an excellent way to uncover the underlying molecular mechanisms linked to disease occurrence, identify novel biomarkers, and drug targets, and ultimately provide further insight for the development of treatment strategies.^5^, ^6^, ^7^ Recently, multiple studies have revealed significant alterations in gut microbiota and gut microbiome‐derived circulating metabolites in CA patients,^8^, ^9^ such as short‐chain fatty acids (SCFAs), bile acids, trimethylamine‐N‐oxide (TMAO), and phenylacetylglutamine (PAGln), on the progression of CVD.^10^, ^11^, ^12^, ^13^

Based on these findings, we propose that a combination of untargeted plasma metabolomics and gut microbiome profiling can provide new insights into the diagnosis of CA in humans. Additionally, several studies have successfully integrated multi‐omics data across various environmental conditions using systems biology approaches.^5^, ^6^, ^7^ For instance, one study including multi‐omics analyses of 161 patients with CAD and 40 healthy controls found that there were notable differences in the gut microbiota and metabolite composition concerning the severity of CAD.^14^ Another multi‐omics study identified a molecular signature for predicting high‐risk atherosclerotic plaques and incident CVD.^15^ Multi‐omics analyses offer a more comprehensive and systematic approach to better understand health and disease. By combining omics data, this approach can identify biomarkers associated with specific diseases, which can be used for disease diagnosis and prognosis.

Therefore, in this study, we aimed to discover the novel diagnostic biomarkers panels associated with CA severity. We recruited a total of 443 participants, including CA patients and healthy controls, from three independent cohorts: discovery, test, and blinded validation. We performed a comprehensive analysis using clinical data, plasma metabolomics, and gut microbiome to distinguish CA patients from healthy controls. Our study revealed that multi‐omics data has high accuracy in identifying CA patients, and in particular, plasma metabolites have the strongest correlation with CA severity. Our findings have important implications for the development of disease classifiers that identify CA severity and may pave the way for the discovery of new diagnostic and prognostic biomarkers for this disease.

## 2 MATERIALS AND METHODS

### 2.1 Study design and population

**FIGURE 1:**
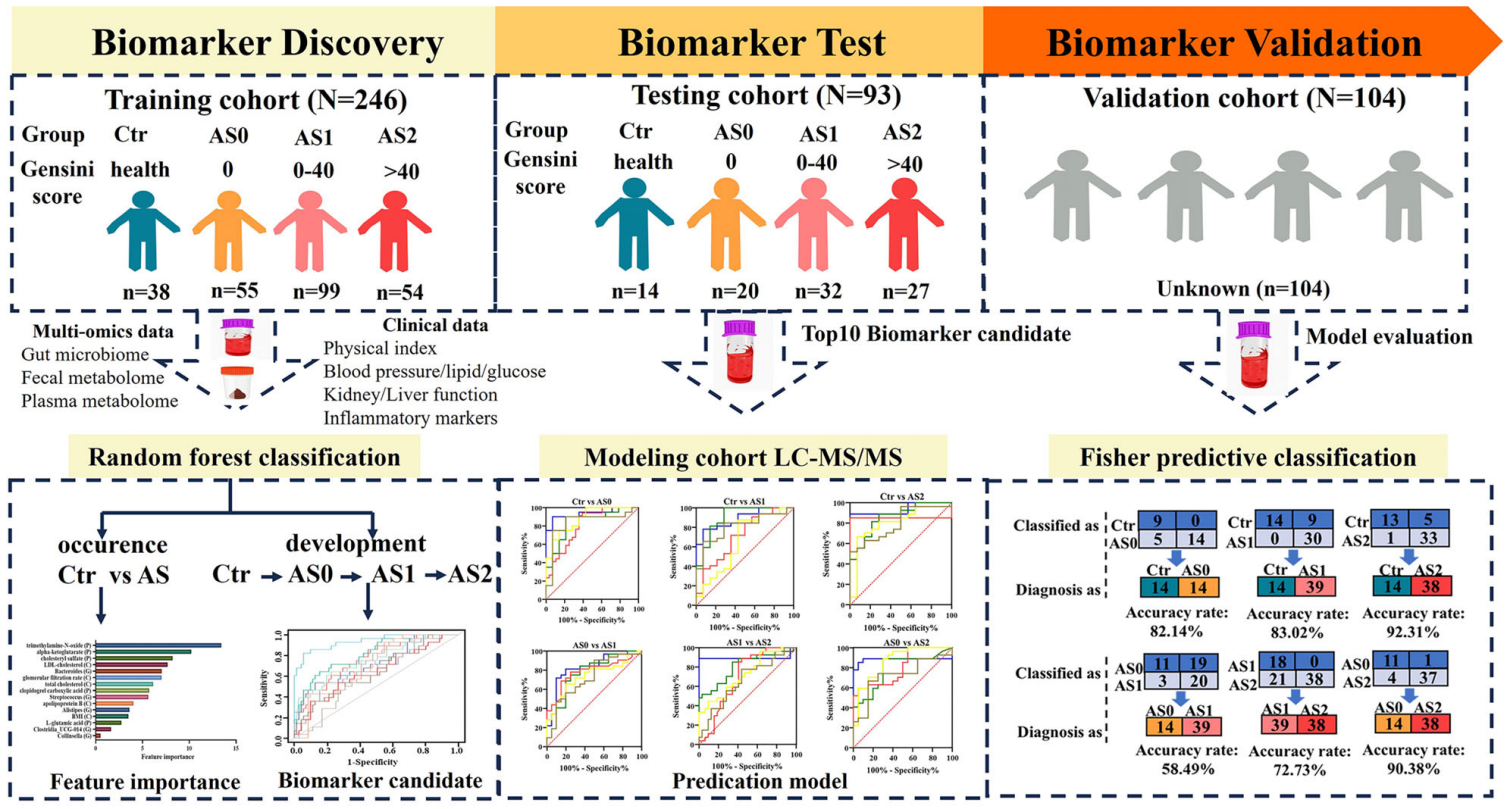
Design of the study. Coronary atherosclerotic patients were identified by coronary angiography and assessed for atherosclerotic burden using the Gensini score (GS), and then divided into atherosclerosis‐free (AS0, GS = 0), mild CA (AS1, GS = 1–40), and severe CA (AS2, GS > 40). For controls, subjects with negative results on the coronary artery or coronary angiography examination, as well as those without clinical symptoms related to cardiovascular disease. The training dataset integrated multi‐omics data to reveal the key features in atherosclerotic pathogenesis and discover biomarker candidates. These candidates were then validated in a plasma test cohort using LC‐MS/MS assay to construct a prediction model for atherosclerotic severity. The validation cohort consisted of 104 plasma samples from healthy controls and CA patients, with 25 molecules targeted for measurement in model evaluation.

A total of 443 participants, including healthy controls (Ctr) and patients, were recruited from Wuxi People's Hospital affiliated with Nanjing Medical University, China. From November 2020 to October 2021, 246 participants were enrolled to form the discovery cohort for initial model development. Subsequently, from November 2021 to May 2023, two independent cohorts were established: a test cohort (*n* = 93) and a validation cohort (*n* = 104), for model refinement and validation, respectively (Figure 1). To address potential confounding effects, multivariate regression analysis was conducted after model development, adjusting for key demographic and clinical variables, including age, sex, body mass index, systolic and diastolic blood pressure, total cholesterol, triglyceride levels, etc. We used CAG which was the gold standard for CA diagnosis and the severity of CA was assessed using the Gensini score (GS).^16^ The GS for grading lumen narrowing was in the Supplementary methods. The CA patients were categorised into three subgroups: atherosclerosis‐free (AS0, GS = 0), mild CA (AS1, GS = 1–40), and severe CA (AS2, GS > 40). For controls, subjects with negative results on the coronary artery or coronary angiography examination, as well as those without clinical symptoms related to CVD, were enrolled. Exclusion criteria included gastrointestinal disorders, chronic liver dysfunction, acute infections, nutritional derangements, malignancies, severe valvular heart diseases, or any other serious medical conditions. Additionally, individuals who had taken probiotics, antibiotics, or antimicrobial agents within the past one month were also excluded. All patients provided written consents to participate in the study, and the study protocol was approved by the Medical Ethics Committee of Jiangnan University (JNU202403RB091).

### 2.2 Sample collection

Blood and fecal samples were collected from all participants before breakfast on the second day after hospitalisation. The participants were instructed to fast for approximately 8–10 h before venipuncture. The blood samples were collected in plastic EDTA tubes and centrifuged at 4000 × *g* for 10 min to separate the plasma. The fecal samples were collected in sterile tubes. All samples were then immediately stored at –80°C and thawed on ice before analysis.

### 2.3 Clinical measurements

Clinical characteristics of all participants, including age, gender, body mass index (BMI), blood pressure, lipid profile (total cholesterol, LDL cholesterol, HDL cholesterol, and triglycerides), fasting glucose levels, etc. were recorded prior to CAG. Specifically, blood pressure was measured using a standard sphygmomanometer after a minimum rest period of 10 min. Lipid profiles and fasting glucose levels were determined using standardised laboratory protocols. All clinical measurements were performed by trained medical professionals in accordance with standardised hospital protocols, and the equipment used was regularly calibrated to ensure accuracy.

### 2.4 16S rRNA gene sequencing analysis

According to the manufacturer's instructions, total DNA was extracted using a Genomic DNA Kit (Tiangen Biotech, Beijing, China). Gel electrophoresis was used to evaluate the DNA sample quality, and DNA purity was determined via 260/280 and 260/230 ratios measured on the Nanodrop. The V3–V4 region of the bacterial 16S ribosomal RNA (rRNA) genes was amplified from the whole genome. The Illumina HiSeq platform was used to run the high‐throughput sequencing on the amplicon libraries. Detailed parameters for data analysis can be found in the Supplementary methods.

### 2.5 Untargeted metabolomics analysis

The untargeted metabolomics protocols were detailed in the Supplementary methods. Briefly, the process encompassed sample preparation, experiment parameters, metabolite identification, and data processing steps. Both plasma and fecal samples from CA patients and healthy controls were subjected to random and blinded testing. Chromatographic analysis was performed on TripleTOF5600+ (AB SCIEX, USA) coupled with an ultra‐performance liquid chromatography (UPLC) system (AB SCIEX ExionLC, USA). To ensure the reliability of the analysis, a quality control sample was integrated after every 15 samples to monitor the consistency of retention times and signal intensities.

### 2.6 Targeted metabolomics analysis of biomarkers

A targeted metabolomics analysis was applied for verifying and validating biomarkers in the validation set. For quantitative determination of the potential markers in the plasma of CA patients, metabolites in plasma were extracted as described above. The potential markers were quantified with multiple reaction monitoring (MRM) mode by QTRAP 5500 (SCIEX, USA) coupled with an UPLC system (Waters, USA). The same liquid chromatography conditions used in the discovery phase were employed for this analysis. Detailed MRM parameters, including ion pairs, declustering potential (DP), collision energy (CE), retention time (RT), and linear correlation equations, are provided in the Supplementary methods.

### 2.7 Biomarker discovery and validation

In the discovery phase, the multi‐omics data, including clinical parameters, gut microbiota composition, and plasma metabolite levels, were integrated and analysed using a combination of statistical and machine learning methods. First, Pearson correlation analysis was employed to identify significant associations between variables across different omics data, revealing potential roles of specific biomarkers in the pathogenesis of CA. To address the potential bias of single‐omics predictions and mitigate overfitting, we applied the random forest algorithm with k‐fold cross‐validation (*k* = 10), which ranked the importance of individual features in distinguishing between healthy and diseased individuals. The top‐ranked features from each omics data were combined to construct multi‐omics biomarkers with predictive accuracy exceeding 90%. For distinguishing disease severity, we employed orthogonal partial least squares‐discriminant analysis (OPLS‐DA), analysis of variance (ANOVA), and fisher's discriminant analysis to evaluate the performance of each omics dataset, revealing that plasma metabolomics was the top‐performing dataset for predicting CA severity. In the testing cohort, the established random forest models were further refined, and the most important variables from plasma metabolomics were used to build a predictive model, which was evaluated using receiver operating characteristic (ROC) analysis. For the validation study, targeted metabolomics was performed on an independent cohort of 104 participants using a targeted metabolomics to quantify candidate biomarkers. Fisher discriminant analysis was then applied to assess the biomarker panel's ability to distinguish CA occurrence and progression. Finally, multivariate logistic regression analysis was performed to evaluate the robustness of the identified biomarkers after adjusting for potential confounding factors. The adjustment covariates included age, sex, body mass index, systolic blood pressure, diastolic blood pressure, total cholesterol, triglyceride levels, etc. This approach ensures that the observed diagnostic performance of the biomarkers is independent of these confounding factors.

### 2.8 Statistical analyses

Comparisons between multiple groups were performed using the Kruskal‒Walli's test and variable importance projection (VIP) scores, and pairwise comparisons were performed using the two‐sided Wilcoxon rank sum test and log2‐fold changes (log2FC) to determine significance. Fisher's exact test was employed for categorical variables. All statistical analyses were performed using R version 4.2.1. A *p* value less than .05 is considered significant.

## 3 RESULTS

### 3.1 Characteristics of the study population

A total of 443 subjects, including healthy controls and patients with CA were recruited in the study to discover biomarker candidates, define potential biomarkers, and validate these biomarkers (Figure 1). Baseline clinical characteristics data in the discovery and testing set are shown in Table 1. The proportion of males was higher among CA patient than in Ctr subjects. In contrast to Ctr participants, CA patients exhibited increased levels of fibrinogen and creatinine, but decreased levels of GFR, TC, and HDL‐C. We compared these different clinical parameters among the CA patients and found that only fibrinogen showed a tendency to increase with CA severity, thus, fibrinogen was the key clinical variable with CA severity (Figure S1E). We conducted principal component analysis (PCA) based on these variables, and the results indicated a division between the Ctr and AS groups (Figure S1F).

**TABLE 1:** Clinical characteristics of the study cohort.

	Discovery set					Testing set				
Characteristics	Ctr (*n* = 38)	AS0 (*n* = 55)	AS1 (*n* = 99)	AS2 (*n* = 54)	*p* Value	Ctr (*n* = 14)	AS0 (*n* = 20)	AS1 (*n* = 32)	AS2 (*n* = 27)	*p* Value
Male, *n* %	47.40%	67.30%	71.70%	63%		42.86%	75.00%	71.88%	81.48%	.072
BMI, kg/m^2^	23.55 ± 3.55	25.19 ± 3.58	24.92 ± 3.10	25.61 ± 3.60	.035	23.62 ± 3.28	24.78 ± 3.28	25.76 ± 3.50	24.97 ± 3.29	.284
Age, year	51.21 ± 9.52	61.18 ± 11.90	63.38 ± 9.56	62.17 ± 9.76	<.001	52.64 ± 8.73	59.00 ± 14.61	60.03 ± 10.71	60.78 ± 11.48	.258
SP, mm Hg	127.29 ± 15.57	134.70 ± 17.87	134.41 ± 19.50	135.02 ± 2.37	.178	127.71 ± 11.74	130.40 ± 14.99	136.06 ± 16.06	137.85 ± 18.74	.168
DP, mm Hg	76.00 ± 11.84	77.51 ± 11.53	75.27 ± 11.11	76.70 ± 9.79	.661	78.07 ± 10.05	74.15 ± 10.41	78.75 ± 8.59	76.93 ± 14.62	.538
TC, mmol/L	4.57 ± .71	3.75 ± .97	3.70 ± 1.11	4.07 ± 1.00	<.001	4.92 ± .91	4.03 ± .98	4.08 ± .96	4.09 ± 1.16	.046
TG, mmol/L	1.33 ± .68	1.57 ± .68	2.84 ± 12.23	2.00 ± 1.62	.681	1.38 ± .59	1.91 ± 1.22	2.06 ± 1.66	1.63 ± .78	.291
LPA, mg/L	154.27 ± 124.97	232.06 ± 262.87	389.40 ± 343.82	368.22 ± 325.22	<.001	240.14 ± 190.46	331.45 ± 288.49	242.48 ± 237.63	275.69 ± 225.46	.583
HDL‐C, mmol/L	1.13 ± .22	1.05 ± .23	.98 ± .21	.97 ± .19	.001	1.23 ± .30	.99 ± .19	.99 ± .22	1.02 ± .28	.021
LDL‐C, mmol/L	2.77 ± .60	2.05 ± .72	2.04 ± .91	2.33 ± .81	<.001	2.76 ± .68	2.19 ± .74	2.16 ± .66	2.28 ± .85	.074
ApoA, mmol/L	1.23 ± .17	1.25 ± .19	1.22 ± .18	1.20 ± .16	.582	1.49 ± .36	1.23 ± .18	1.22 ± .22	1.19 ± .25	.003
Apo B, mmol/L	.75 ± .15	.65 ± .19	.65 ± .20	.72 ± .21	.009	.76 ± .16	.65 ± .15	.67 ± .19	.71 ± .21	.334
FBG, mmol/L	4.57 ± .45	5.04 ± .88	5.09 ± 1.05	5.19 ± .99	.011	4.84 ± .85	4.88 ± .53	5.28 ± 1.92	5.48 ± 2.72	.642
Creatinine, µmol/L	64.58 ± 13.67	72.29 ± 13.70	76.20 ± 15.76	71.70 ± 13.10	.001	61.08 ± 21.71	74.47 ± 13.32	74.13 ± 15.98	79.62 ± 16.66	.012
BUN, mmol/L	5.3 ± 1.19	5.98 ± 1.89	5.85 ± 1.50	5.99 ± 1.47	.14	4.91 ± 1.14	5.45 ± 1.07	5.35 ± 1.03	5.94 ± 1.11	.03
UA, µmol/L	320.92 ± 74.89	377.44 ± 89.23	373.78 ± 94.10	376.24 ± 95.35	.01	291.57 ± 67.17	349.32 ± 71.18	354.99 ± 109.02	351.83 ± 62.18	.121
GFR, mL/min	106.06 ± 12.10	91.10 ± 14.47	86.90 ± 13.64	90.51 ± 11.65	<.001	102.54 ± 15.44	90.98 ± 16.15	90.78 ± 14.74	86.77 ± 15.28	.024
ALT, U/L	20.46 ± 16.17	22.40 ± 11.45	22.51 ± 11.46	25.29 ± 13.98	.395	14.24 ± 6.69	28.94 ± 28.70	27.63 ± 20.28	24.98 ± 11.57	.084
AST, U/L	21.74 ± 13.28	23.49 ± 16.01	24.45 ± 12.77	26.55 ± 15.01	.08	17.69 ± 3.40	25.15 ± 17.32	24.97 ± 13.53	24.27 ± 13.18	.279
ALP, U/L	65.29 ± 18.08	75.09 ± 19.26	74.90 ± 18.03	78.22 ± 32.30	.047	66.00 ± 19.38	77.80 ± 19.93	83.31 ± 22.52	77.70 ± 18.87	.114
GGT, U/L	26.11 ± 15.72	30.98 ± 15.77	30.69 ± 17.14	29.79 ± 15.11	.467	17.00 ± 6.76	32.65 ± 18.07	32.31 ± 20.99	34.15 ± 20.75	.05
TP, g/L	66.10 ± 5.43	65.54 ± 5.20	65.34 ± 4.17	67.09 ± 4.65	.162	68.13 ± 5.77	65.87 ± 5.23	64.90 ± 4.49	65.14 ± 4.42	.197
A/G	1.52 ± .18	1.51 ± .20	1.50 ± .21	1.45 ± .22	.338	1.52 ± .17	1.58 ± .20	1.52 ± .19	1.58 ± .29	.679
TB, µmol/L	13.53 ± 5.45	14.81 ± 4.83	14.35 ± 5.92	13.66 ± 4.86	.594	15.29 ± 4.52	15.77 ± 5.02	13.52 ± 4.19	13.86 ± 4.84	.289
hs‐CRP, mg/L	1.64 ± 1.10	1.72 ± 1.32	2.10 ± 1.61	2.99 ± 2.33	.048	1.48 ± 1.14	2.10 ± 1.75	2.48 ± 2.06	2.81 ± 2.39	.678
TLC, 10^12^/L	5.96 ± 1.31	6.68 ± 1.70	6.89 ± 1.86	6.91 ± 1.40	.022	6.68 ± 2.31	6.67 ± 1.70	7.25 ± 2.61	6.79 ± 1.73	.731
Fibrinogen, g/L	2.41 ± .35	2.69 ± .50	2.82 ± .60	2.90 ± .63	<.001	2.76 ± .61	2.91 ± .54	2.98 ± .51	3.43 ± .83	.004
D‐Dimer, µg/L	103.41 ± 59.74	115.91 ± 93.75	122.77 ± 111.17	114.83 ± 81.45	.781	77.38 ± 26.30	104.45 ± 81.32	82.66 ± 38.31	120.96 ± 81.29	.081

Abbreviation: SP: systolic pressure; DP: diastolic pressure; TC: total cholesterol; TG: triglyceride; LPA: lipoprotein A; HDL‐C: HDL‐cholesterol; LDL‐C: LDL–cholesterol; ApoA: apolipoprotein A; ApoB: apolipoprotein B; FBG: fasting blood‐glucose; BUN: blood urea nitrogen; UA: uric acid; GFR: glomerular filtration rate; ALT: alanine transaminase; AST: aspartate transaminase; ALP: alkaline phosphatase; GGT: gamma‐glutamyl transferase; TP: total protein; A/G: albumin/globulin; TB: total bilirubin; TLC: total leukocyte count.

### 3.2 Gut microbiome composition and fecal metabolites of coronary atherosclerosis patients

**FIGURE 2:**
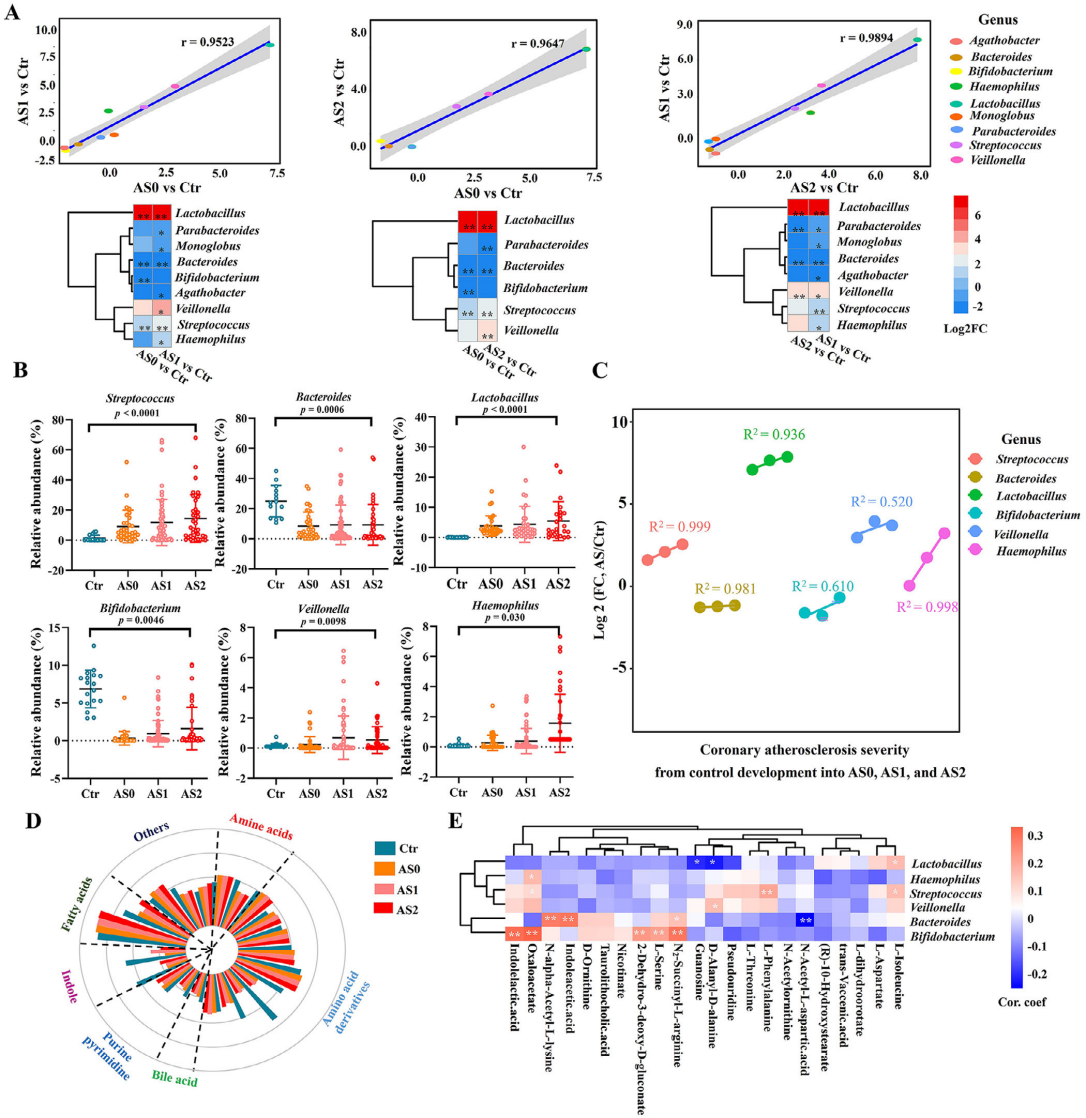
Changes in gut microbiome and fecal metabolites. (A) Scatter plot with a linear regression line and heatmap depicting Log2FC‐based alterations of the significantly different genus in the gut microbiome with different severity of coronary atherosclerosis, *p* < .05. Log2FC: log2 (fold change). (B) Differential analysis of the gut microbiota at the genus level. (C) Regression curve depicting the significantly different genus with different severity of coronary atherosclerosis, from control development into AS0, AS1, and AS2. (D) Changes and differences in fecal metabolites. (E) Correlation between gut microbiota and fecal metabolites (Spearman's correlation test, *: *p < *.05, **: *p < *.01).

Using 16 S rRNA gene sequencing to study the microbial composition in the gut and explore the interactions between the host and microbiome in subjects with varying degrees of CA. The microbes of AS groups had no significant differences concerning alpha diversity as determined by the Chao, Simpson, Shannon, and Ace indices compared with the healthy controls (Figure S2A). In addition, the gut microbiota in the AS and control groups was dominated by four abundant phyla with significant differences in *Bacteroidota* and *Actinobacteriota* between the groups. Meanwhile, the abundance of *Bacteroidales*, *Lactobacillales*, and *Bifidobacteriales* at the order level, as well as *Bacteroidaceae*, *Streptococcaceae*, and *Bifidobacteriaceae* at the family level was significantly different between AS and Ctr groups (Figure S2C). The genus exhibiting significant changes between the AS0, AS1, or AS2 groups and the Ctr group was determined using the Wilcoxon signed‐rank test (*p* < .05). Then, we performed a linear regression analysis for these significantly altered genera between each AS group versus the Ctr group. As presented in Figure 2A, the change of genus showed a positively correlated tendency between the AS0 and AS1 groups, AS0 and AS2 groups, and AS1 and AS2 groups, suggesting a fraction of similar changes in the gut microbe composition, when CA develops into the severe stage.

Based on the differential analysis of the gut microbe, we found that the abundances of individual genera of *Lactobacillus*, *Streptococcus*, *Veillonella*, and *Haemophilus* were significantly increased, and only *Bacteroides* and *Bifidobacterium* were significantly reduced between AS groups versus Ctr group (Figure 2B). To further explore the relationship between these significantly different genus and CA severity, we conducted a regression curve analysis and found that the abundance of *Streptococcus, Bacteroides, Lactobacillus* and *Haemophilus* were correlated with the severity of CA (*R* ^2 ^> .9). Specifically, *Streptococcus, Lactobacillus and Haemophilus* showed a positive correlation with the progression of CA, and *Bacteroides* exhibited a negative correlation (Figure 2C). Collectively, these findings suggest that alterations in the abundance of certain microbial genera may be associated with the progression of AS.

We performed fecal metabolomic analyses to assess functional characteristics of gut microbiota and metabolites (Dataset S1), of which 21 metabolites showed significant (*p *< .05) differences in at least one of the stages compared to healthy controls (Figure 2D) (Dataset S1). Indole, amino acids, and its derivatives were the focuses of attention, and compared with the Ctr group, the levels of indoleacetic acid, indolelacetic acid, N‐alpha‐acetyl‐L‐lysine, N2‐succinyl‐L‐arginine, *L*‐serine, and 2‐dehydro‐3‐deoxy‐d‐gluconate were decreased (Figure 2D). According to the fecal metabolomic and 16S analyses, indoleacetic acid, N2‐succinyl‐L‐arginine, and *L*‐serine were positively correlated with *Bifidobacterium* (*p *< .05), and indoleacetic acid and N‐alpha‐acetyl‐L‐lysine were positively correlated with *Bacteroides* (*p *< .05) (Figure 2E). Our data indicate that the gut microbe and fecal metabolites change with the progression of CA.

### 3.3 Metabolomics alterations in the plasma of coronary atherosclerosis patients

**FIGURE 3:**
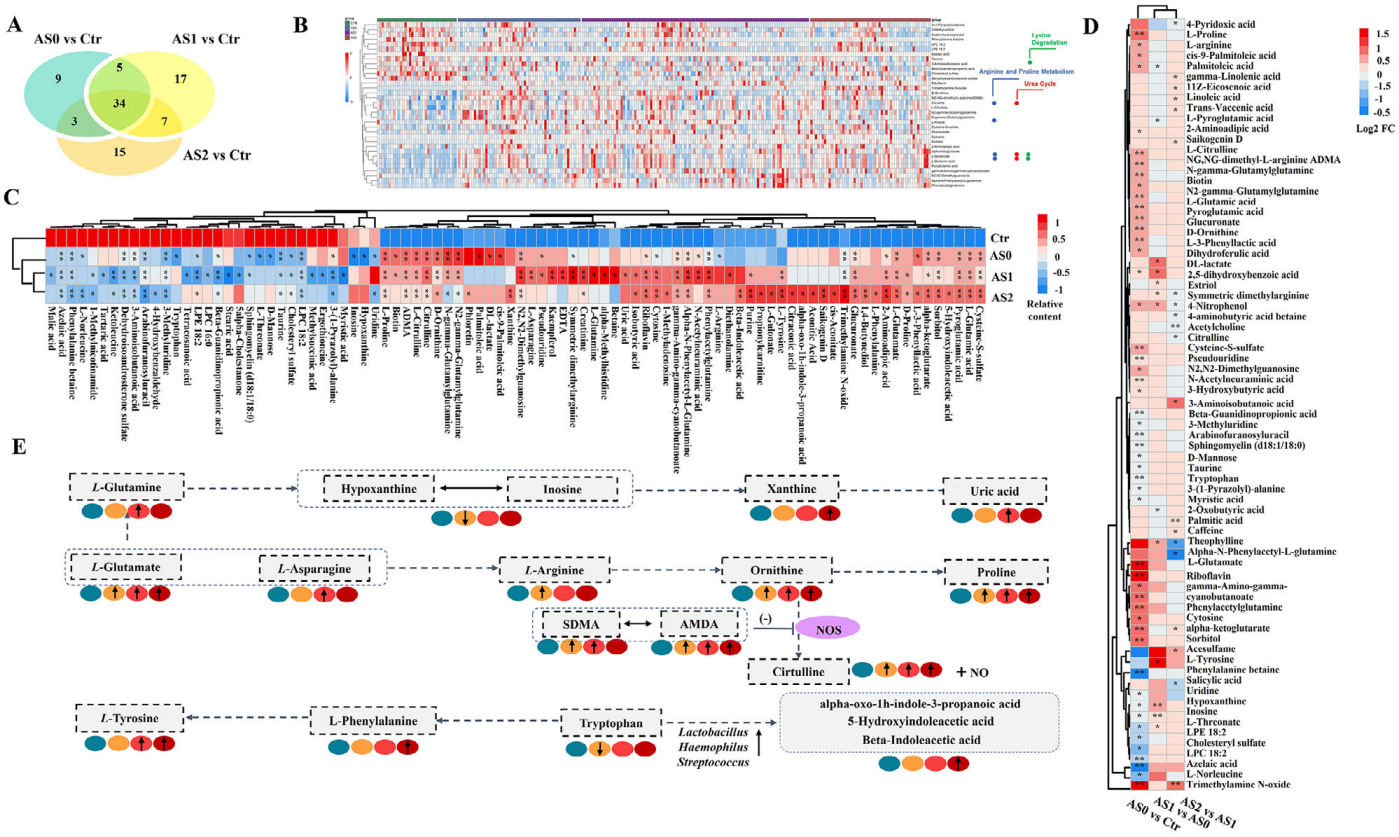
Comparison of the plasma metabolites. (A) Venn diagram showing the overlaps among altered metabolites (*p* < .05, compared Ctr vs. AS0, Ctr vs. AS1, and Ctr vs. AS2). (B) Heatmap showing significantly different metabolites in all AS groups compared Ctr. (C) Heatmap showing relative content of significantly different metabolites when comparing Ctr vs. AS0, Ctr vs. AS1, and Ctr vs. AS2, *: *p* < .05, **: *p* < .01. (D) Heatmap showing significantly different metabolites compared coronary atherosclerosis severity, Log2FC: log2 (fold change), *: *p *< .05, **: *p* < .01. (E) Enrichment pathway related to coronary atherosclerosis severity.

To identify the plasma metabolome features of CA patients, untargeted metabolome profiles were generated by LC‐MS/MS (Dataset S2). We discovered that there were notable differences in plasma metabolites between CA patients and healthy controls based on the OPLS‐DA models of metabolite profiling data (Figure S3A). Then, we identified 51, 63, and 59 metabolites significantly different in the AS0, AS1, AS2 groups compared with Ctr respectively (*p *< .05, Dataset S2 (Figure 3A). Of these metabolites, 34 of them are common in all AS stages compared with Ctr, and we plotted a heat map to display all these 34 significantly different metabolites in the AS versus Ctr in Figure 3B. We found the plasma level of TMAO, PAGln was significantly higher in AS group (Figure 3B). TMAO and PAGln, both metabolic products of the gut microbe, are associated with an increased risk of CVD, diabetes, and cancer.^10^, ^13^ And among them, 9 metabolites were significantly different only in AS0 versus Ctr, 17 metabolites significantly differed only in AS1 versus Ctr, and 15 metabolites significantly differed only in AS2 versus Ctr (Figure 3C). On the other hand, we also compared the different metabolites with CA severity (Figure S3B), we found that plasma level of 50 metabolites significantly differed in AS0 versus Ctr, 13 metabolites significantly differed in the AS1 versus AS0, and 20 metabolites significantly differed in the AS2 versus AS1 (Figure 3D). The findings revealed that the metabolite profiles of patients with CA differed markedly from those of healthy controls, and that the metabolite levels might further shift with CA severity.

Notably, we found that purine metabolism, tryptophan metabolism, and *L*‐arginine biosynthesis were correlated with the development of CA (Figure 3E). In purine metabolism, we observed that the plasma level of hypoxanthine and inosine were significantly decreased in AS0, xanthine was significantly increased in AS2, and uric acid was significantly increased in AS1 (Figure 3E). In tryptophan (Trp) metabolism, Trp was significantly decreased in AS0, and Trp metabolites such as alpha‐oxo‐1h‐indole‐3‐propanoic acid, 5‐hydroxyindoleacetic acid, and beta‐indoleacetic acid were significantly increased in AS2 (Figure 3E). In *L*‐arginine biosynthesis, we also found that the plasma level of *L*‐arginine was significantly increased in AS0, ornithine and proline were significantly increased in all AS groups. Asymmetric dimethylarginine (ADMA) was significantly increased in all AS groups, and symmetric dimethylarginine (SDMA) was significantly increased in AS0 and AS1 groups (Figure 3E).

### 3.4 Prediction of coronary atherosclerosis occurrence based on multi‐omics data

**FIGURE 4:**
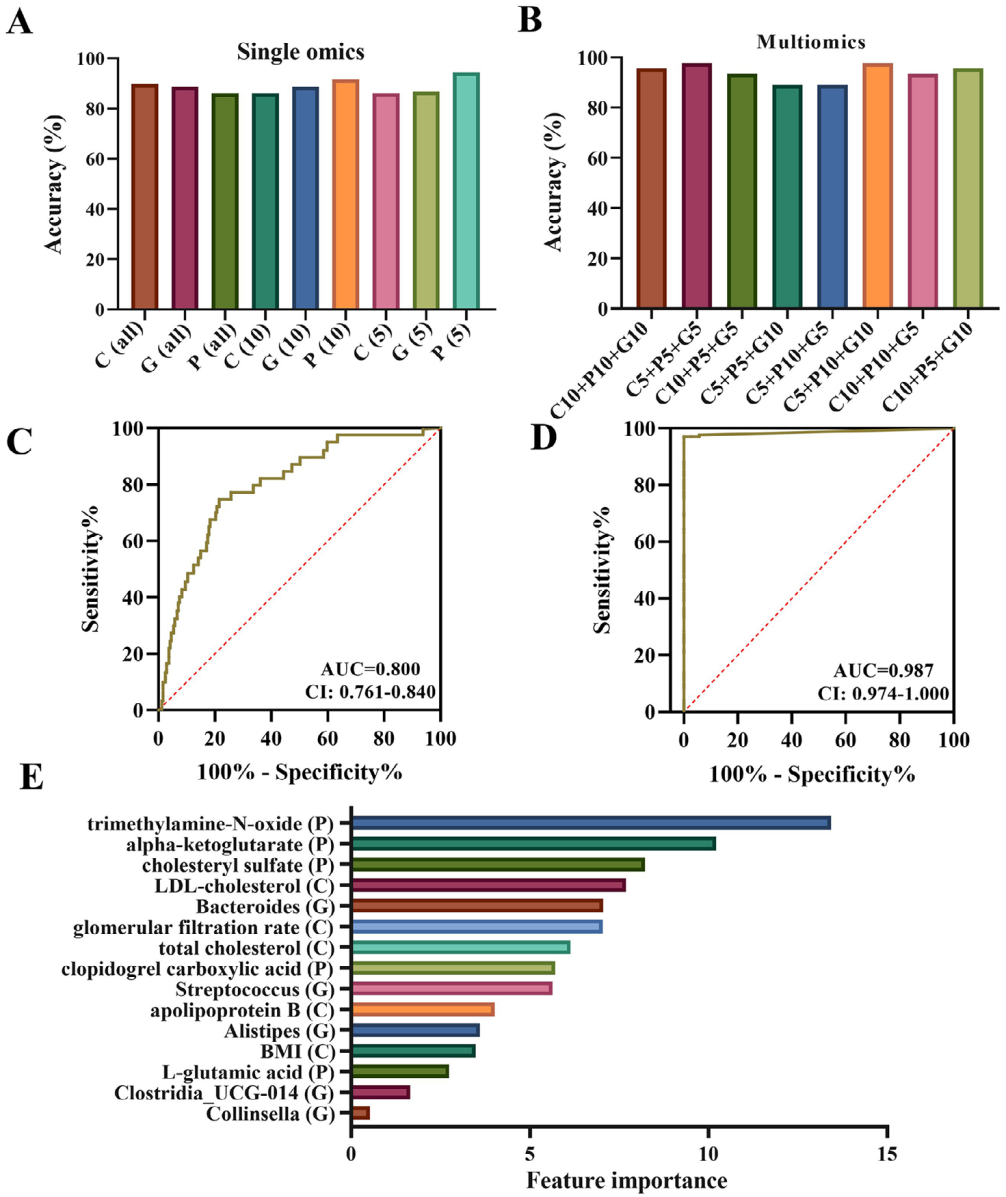
Accuracy score of random forest classification algorithm for predicting the class using omics data. (A) Single omics. (B) Multi‐omics combination of top features from each omics. (C) Receiver operating characteristic (ROC) curve for prediction of coronary atherosclerosis based on Top 5 feature from plasma metabolites. (D) ROC curve for prediction of coronary atherosclerosis based on combination of P (5), C (5), and G (5). (E) Top 15 features from the model with the highest accuracy, P (5) + C (5) + G (5).

For the training cohort, we built a random forest machine learning model based on plasma metabolites, gut microbe, and clinical features (single/multi‐omics data) and discovered the key features associated with CA. First, we made use of all data from each omics (Figure 4A). We discovered that the plasma metabolomics data were the best‐performing dataset for the prediction of CA severity, with an area under the curve (AUC) of .800 and a prediction accuracy of over 80%, (Figure 4C). On the other hand, we discovered that the gut microbiota data performed the worst, with an AUC of .638 (Figure S5B). Subsequently, we experimented with various combinations of the top five or ten features from each omics dataset (Figure 4B). We found that the top five features in clinical (C5), plasma metabolomics (P5), and gut microbiota (G5) produced an accuracy of 97.83% (AUC = .987) (Figure 4D). The top five features in each category—clinical parameters (BMI, total cholesterol, LDL‐cholesterol, apolipoprotein B, and glomerular filtration rate), plasma metabolomics (alpha‐ketoglutarate, cholesteryl sulphate, L‐glutamic acid, TMAO, and clopidogrel carboxylic acid), and gut microbiota (*Alistipes*, *Collinsella*, *Clostridia_UCG‐014*, *Streptococcus*, and *Bacteroides*)—may serve as candidate biomarkers for the occurrence of CA (Figure 4E).

To determine whether the single/multi‐omics data can be regarded as biomarkers for distinguishing CA severity, we studied the contribution of each omics data to the distinguish CA severity. In addition, both the OPLS‐DA model and ANOVA results showed that plasma metabolomics was the top‐performing dataset in the prediction of CA severity (Figure S6A). The results of Fisher discriminant analysis also showed that plasma metabolites contributed more to the calculation of subgroups (Figure S6B) (Dataset S4).

Then random forest models were constructed to classify different stages of CA based on plasma metabolites, and ROC curves were used to test the classification. We could accurately distinguish CA patients from healthy controls, as indicated by the AUC, which had a value up to .971 separated Ctr from AS0, .964 separated Ctr from AS1, .991 separated Ctr from AS2 (Figure S7A and B).

In the subgroup comparisons, we considered AS0 versus AS1 for plaque formation and found that AS0 and AS1 were discriminated by 13 plasma metabolites with an AUC of .682, 20 plasma metabolites classified AS1 from AS2 with an AUC of .757 and 15 plasma metabolites classified AS0 from AS2 with an AUC of .833 (Figure S7C and D).

### 3.5 Defining potential metabolic biomarkers for coronary atherosclerosis severity

**FIGURE 5:**
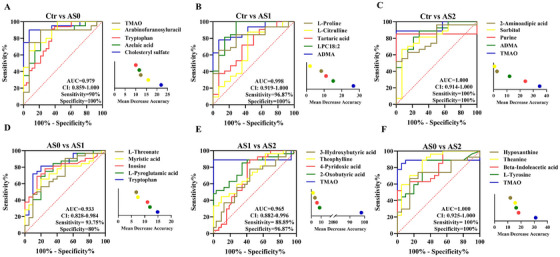
Target plasma metabolites identify high‐performing diagnostic and prognostic biomarkers for coronary atherosclerosis. Receiver operating characteristic (ROC) analysis to discriminate of Ctr, AS0, AS1, and AS2 groups. A: Ctr vs. AS0, AUC = .979. B: Ctr vs. AS1, AUC = .998. C: Ctr vs. AS2, AUC = 1.000. D: AS0 vs. AS1, AUC = .933. E: AS1 vs. AS2, AUC = .965. F: AS0 vs. AS2, AUC = 1.000.

Following the discovery phase, we enrolled another independent testing cohort that satisfied the same inclusion and exclusion criteria (Table 1). Based on the plasma metabolomics data from the testing study, we used the established random forest models to further screen biomarker panels, which can effectively distinguish subgroups of CA, and ROC curves were used to test the classification. Given the high degree of variability in drug usage among patients, we excluded drugs from the final model establishment to ensure the robustness and generalisability of our findings. We mainly established six models, namely, Ctr versus AS0, Ctr versus. AS1, Ctr versus AS2, AS0 versus AS1, AS1 versus AS2, and AS0 versus AS2. Consistently, the features of cholesteryl sulphate, azelaic acid (AA), Trp, arabinofuranosyluracil, and TMAO can help distinguish Ctr versus AS0 (AUC .979; Figure 5A); the features of ADMA, LPC18:2, tartaric acid, *L*‐citrulline, and *L*‐proline can help distinguish Ctr versus AS1 (AUC .998; Figure 5B); the features of TMAO, ADMA, purine, sorbitol, and 2‐aminoadipic acid (2AA) can help distinguish Ctr versus AS2 (AUC 1.000; Figure 5C); the features of Trp, L‐pyroglutamic acid (PGA), inosine, myristic acid, and *L*‐threonate can help distinguish AS0 versus AS1 (AUC .933; Figure 5D); the features of TMAO, 2‐oxobutyric acid, 4‐pyridoxic acid, theophylline, and 3‐hydroxybutyric acid can help distinguish AS1 versus AS2 (AUC .965; Figure 5E); the features of TMAO, *L*‐tyrosine, beta‐indoleacetic acid, theanine, and hypoxanthine can help distinguish AS0 versus AS2 (AUC 1.000; Figure 5F). The sensitivity, specificity, and AUC for both individual and combined biomarker panels are detailed in Table S1. Overall, the metabolic features captured by the classifier highlighted its great potential for the detection of CA stage.

### 3.6 Validation of the biomarker panel for coronary atherosclerosis severity

**FIGURE 6:**
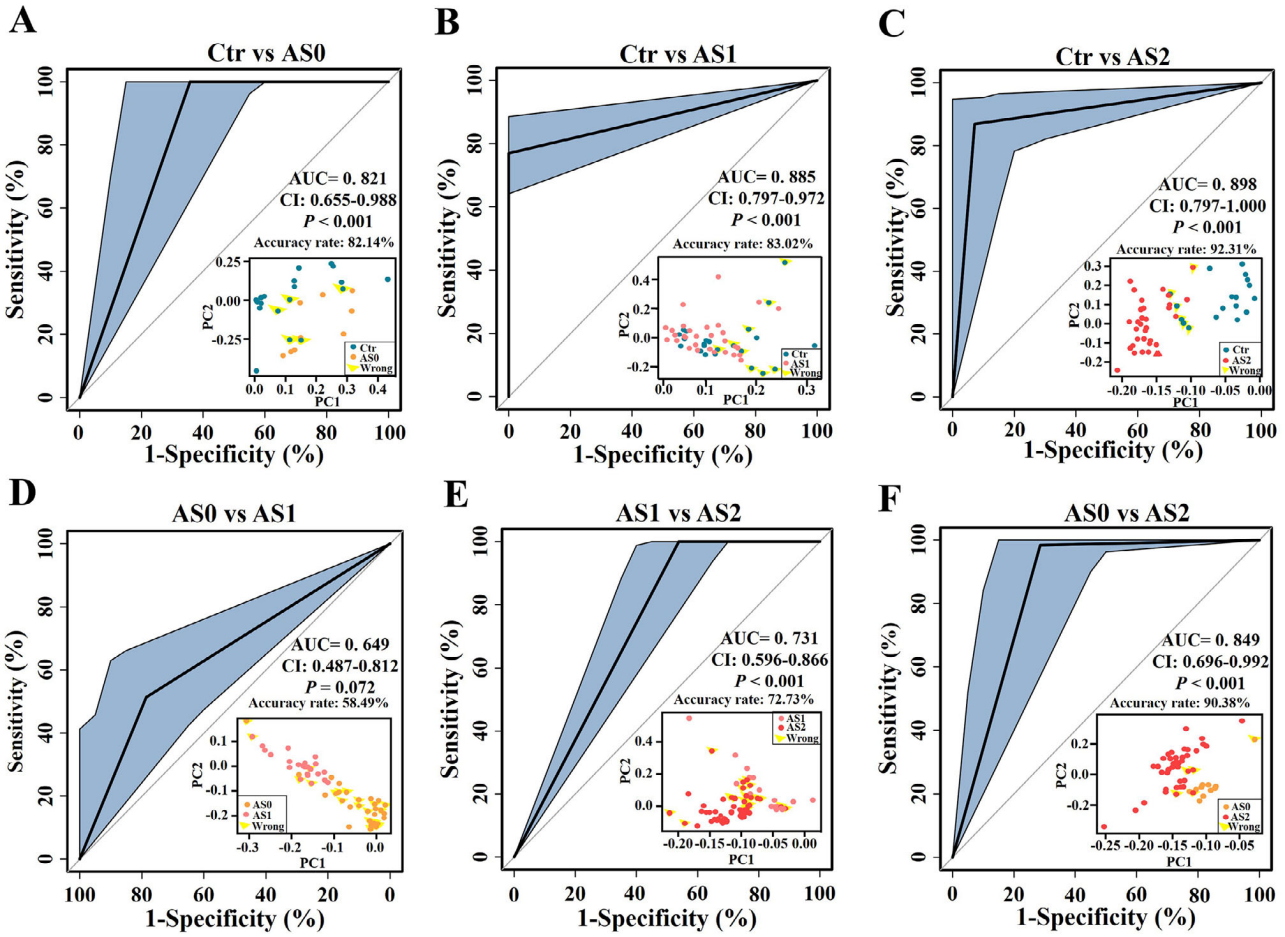
Validation performance in the blinded validation cohort. Receiver operating characteristic (ROC) curve analysis for the predictive power of validated signatures in a new plasma cohort of 104 unknown participants. Samples classified into the wrong group were labelled. A: Ctr vs. AS0, AUC = .821, accuracy rate = 82.14%. B: Ctr vs. AS1, AUC = .885; accuracy rate = 83.02%. C: Ctr vs. AS2, AUC = .898, accuracy rate = 92.31%. D: AS0 vs. AS1, AUC = .649, accuracy rate = 58.49%. E: AS1 vs. AS2, AUC = .731, accuracy rate = 72.73%. F: AS0 vs. AS2, AUC = .849, accuracy rate = 90.38%.

To validate the prediction power of the above‐constructed metabolite panel in plasma selected as potential indicators to distinguish CA patients, we performed the targeted metabolomics analysis with plasma samples from an independent set (validation cohort, Table S2) of 104 participants based on the criteria that were used during the discovery phase. Potential biomarkers discovered in the testing cohort were targeted and quantified to evaluate the accuracy of prediction in the validation cohorts of unknown samples (Dataset S5). The prediction was evaluated by the ROC analysis, as shown in Figure 6A, the combination of these metabolite panels distinguished Ctr versus AS0 with an AUC of .821 (sensitivity 100%, specificity 64.29%, and accuracy rate 82.14%) (Figure 6A); Ctr versus AS1 with AUC of .885 (sensitivity 76.92%, specificity 100%, and accuracy rate 83.02%) (Figure 6B); Ctr versus AS2 with AUC of .898 (sensitivity 86.84%, specificity 92.86%, and accuracy rate 92.3%) (Figure 6C); AS0 versus AS1 with AUC of .649 (sensitivity 51.28%, specificity 78.57%, and accuracy rate 58.49%) (Figure 6D); AS1 versus AS2 with AUC of .731 (sensitivity 100%, specificity 46.15%, and accuracy rate 72.73%) (Figure 6E); AS0 versus AS2 with AUC of .849 (sensitivity 97.37%, specificity 71.43%, and accuracy rate 90.38%) (Figure 6F). AS samples were correctly differentiated from controls with high and consistent accuracy, and most samples could be classified into the right group. While the diagnostic ability of AS0 differs from AS1 was not satisfactory. After adjusting various comorbidities factors (age, sex, body mass index, systolic pressure, diastolic pressure, total cholesterol, triglyceride, etc.), we observed that 90% of these biomarkers remained significant (*p* < .05). The adjusted odds ratios with their corresponding 95% confidence intervals (CIs) and *p*‐values are reported in Table S3. These results demonstrated that CA severity can be distinguished using the above‐constructed biomarker panels, further highlighting their potential utility in clinical practice, even in diverse patient populations.

## 4 DISCUSSION

Our study provides novel insights into the systemic metabolic and microbial alterations associated with CA, offering a comprehensive understanding of its pathophysiological mechanisms and potential diagnostic applications. These findings build upon emerging evidence linking gut microbial dysbiosis to CVDs pathogenesis, while simultaneously advancing our knowledge of plasma metabolite profiles in CA progression. By integrating multi‐omics approaches, we identified distinct signatures in both the gut microbiome and plasma metabolome that may serve as robust biomarkers for CA severity.

We observed significant compositional shifts in the gut microbiome of CA patients, characterised by increased abundances of pro‐inflammatory genera (*Lactobacillus*, *Streptococcus*, *Veillonella* and *Haemophilus*) and decreased levels of beneficial taxa (*Bacteroides* and *Bifidobacterium*). Notably, the positive correlation between *Streptococcus*, *Lactobacillus*, and *Haemophilus* abundance and CA severity suggests their potential role in disease progression. The enrichment of *Veillonella* and *Haemophilus*, known for their pro‐inflammatory properties through lipopolysaccharide production,^17^ coupled with the reduction of *Bifidobacterium*, which exerts beneficial metabolic effects,^18^ underscores the critical role of gut microbiota in cardiovascular health.

Metabolic profiling identified a robust plasma biomarker panel (AUC: .979–1.000) capable of distinguishing CA patients from healthy controls with over 80% accuracy in validation cohorts. The biomarker panel comprised multiple metabolites with distinct pathophysiological roles, including cholesteryl sulphate, azelaic acid (AA), Trp, TMAO, ADMA, LPC18:2, *L*‐citrulline, purine, and 2‐aminoadipic acid (2AA). These metabolites exhibit multifaceted cardiovascular effects, including modulation of plaque formation, endothelial dysfunction, and coronary artery calcification. For instance, cholesteryl sulphate deficiency promotes atherosclerotic plaque formation,^19^ while AA, a product of linoleic acid oxidation, may attenuate the progression of AS.^20^ Alterations in Trp metabolism, such as the elevated kynurenine‐to‐Trp ratio, are associated with established CVD risk factors and subclinical atherosclerosis markers.^21^ LPC18:2 binds C‐reactive protein and inhibit its pro‐atherogenic effects,^22^ whereas *L*‐ citrulline, a precursor of nitric oxide, exerts cardiovascular benefits.^23^ Additionally, purine derivatives, including xanthine, hypoxanthine, and uric acid, modulate uremic toxins and are implicated in CVD‐related traits.^24^ We observed a significant decrease in plasma purine concentration in AS2 compared to Ctr, while xanthine, a purine‐derived metabolite, was significantly elevated. These findings underscore the diagnostic and therapeutic potential of the biomarker panel. Furthermore, significant decreases in Trp, AA, LPC18:2, *L*‐citrulline, *L*‐proline, and purine concentrations were observed in CA patients (Figure S8A), reinforcing their utility as diagnostic biomarkers. Our results also highlight the emerging role of gut microbiota‐derived metabolites in cardiovascular pathology. TMAO, a gut microbial metabolite, was significantly elevated in CA patients, consistent with large cohort studies linking high plasma TMAO levels to an increased incidence of major adverse cardiovascular events.^10^ Additionally, ADMA, an endogenous inhibitor of NO synthesis, plays a critical role in f endothelial dysfunction and serves as a marker of oxidative stress.^25^ Similarly, 2AA was strongly associated with the extent of coronary artery calcification, a marker of subclinical AS risk.^26^ Elevated plasma concentrations of ADMA and 2AA in CA patients (Figure S8A) further support their roles in endothelial dysfunction^27^ and coronary artery calcification,^26^ respectively. Collectively, these findings highlight the multifaceted roles of the identified metabolites in CA pathogenesis and their potential as diagnostic and therapeutic targets.

The plasma biomarker panels effectively predicted the severity of CA with AUC values exceeding .933. Key metabolites contributing to CA severity prediction included Trp, PGA, inosine, myristic acid and *L*‐threonate, TMAO, 2‐oxobutyric acid, 4‐pyridoxic acid, theophylline, 3‐hydroxybutyric, L‐tyrosine, beta‐indoleacetic acid, theanine, and hypoxanthine. Specifically, the progression from AS0 to AS1, characterised by plaque formation, was associated with Trp, PGA, inosine, myristic acid, and L‐threonate (Figure 5D). AS, a lipoprotein‐driven disease, involves intimal inflammation, necrosis, fibrosis, and calcification, leading to plaque formation at specific arterial sites.^28^ Calcific vasculopathy, prevalent in advanced lesions, promotes arterial stiffness and plaque development.^29^ A recent study by Ouyang et al. highlighted the role of tryptophan metabolism in vascular calcification, showing a negative correlation between the kynurenine‐to‐Trp ratio and atherosclerotic calcification progression.^30^ Consistent with this, we observed increased Trp levels (Figure S8B), which may promote plaque formation. PGA, linked to glutathione metabolism, showed reduced plasma levels (Figure S8B), suggesting downregulated glutathione turnover, a known factor in CVD development. Inosine, a purine nucleoside with coronary vasodilatory and antiplatelet effects, significantly increased during plaque formation (Figure S8B), potentially indicating a self‐regulatory response.^31^ Elevated myristic acid levels, associated with increased HDL, were observed in CA patients, though HDL decreased during plaque formation, aligning with previous findings.^28^ *L*‐threonate, linked to AS severity through ROS and pro‐inflammatory cytokine production, also increased (Figure S8B), further supporting its role in plaque development.^32^ The progression from AS0 to AS1 involves dynamic plaque formation and rupture processes, which remain poorly understood at the metabolic level. Our study provides insights into these molecular mechanisms. In validation sets, diagnostic accuracy was 58.49%, 72.73%, and 90.38% for different stages. While AS0 the performance of AS0 and AS1 individuals was not satisfactory, this likely reflects the complex and dynamic nature of early plaque formation.

Our approach offers two translational advantages over invasive CAG and imaging modalities: (1) Early diagnostic capability: The panel demonstrated high diagnostic performance (AUC > .933) in distinguishing CA severity, with superior accessibility and cost‐effectiveness. After adjusting for comorbidities, 90% of the biomarkers remained significant. (2) Cost‐effectiveness: This method eliminates radiation exposure and specialised equipment requirements, making it more accessible for widespread clinical adoption. However, to translate these findings into clinical practice, several critical steps must be addressed, including conducting large‐scale, multicentre trials to ensure generalisability and reproducibility, obtaining regulatory approval from agencies, and developing standardised protocols for sample collection and assay performance, addressing practical barriers like healthcare provider training and workflow integration. Importantly, the implementation of biomarker‐based diagnostics raises ethical and accessibility considerations, particularly regarding cost and equity. To ensure equitable access, strategies must be developed to minimise testing costs and address potential disparities in healthcare resource availability, especially in low‐resource settings. By systematically addressing these challenges, this biomarker‐based approach can be effectively integrated into clinical practice, improving patient outcomes and reducing healthcare disparities globally.

This study identified significant changes in metabolite levels during CA progression and pointed out the biomarker for assessing CA severity. However, several limitations should be noted. The hospital‐based cohort, primarily comprising symptomatic CA patients referred for CAG, may limit generalisability, as asymptomatic individuals were excluded. Additionally, a significant proportion of participants were on lipid‐lowering therapy, potentially influencing metabolite profiles and confounding associations. Future studies should stratify participants by treatment status or include more untreated individuals to better understand the natural progression of CA. The exclusion of patients with gastrointestinal disorders, chronic liver dysfunction, acute infections, and other comorbidities may also restrict broader applicability. Future research should aim to include a more diverse range of subjects, incorporating individuals with varying risk levels and comorbid conditions to enhance external validity. The external standard method used for metabolite quantification may not fully account for sample matrix effects, suggesting the incorporation of internal standards or alternative methods to improve accuracy. Lastly, the lack of long‐term follow‐up data limits risk stratification potential, underscoring the need for prospective studies with extended follow‐up to validate the prognostic value of these biomarkers and ensure the robustness and applicability across diverse populations.

In conclusion, this study reveals a correlation between significant changed gut microbiota and plasma metabolites with the occurrence and development of CA. The biomarker panel demonstrated high diagnostic accuracy in distinguishing CA occurrence and severity, even after adjusting for various comorbidities, with 90% of the biomarkers remaining significant. These findings provide new insights into novel biomarkers for assessing CA severity and highlight their potential for clinical diagnosis. However, further investigation is needed to elucidate the underlying mechanisms of key metabolites and rigorously validate their clinical utility in larger, diverse populations, which are essential steps to translate these findings into clinical applications and improve patient outcomes.

## AUTHOR CONTRIBUTIONS

Y.J.X., R.X.W, and Y.F.L. contributed to the conceptualisation and design of the study. M.X.H. and D.X.W. contributed to data analysis. M.X.H., D.X.W., J.C.S., A.Y.L., X.X.Z., Y.L.G., Y.H., and Y.Z. contributed to data collection. M.X.H. contributed to the manuscript writing. Y.J.X., R.X.W, and Y.F.L. wrote, reviewed, and edited the manuscript. All authors read, revised, and approved the final draft of the manuscript.

## CONFLICT OF INTEREST STATEMENT

The authors declare no competing interests.

## ETHICS STATEMENT

The study was approved by the Medical Ethics Committee of Jiangnan University (JNU202403RB091). All participants provided written informed consent prior to their participation.

## Supporting information



Supporting Information

Supporting Information

Supporting Information

Supporting Information

Supporting Information

Supporting Information

## Data Availability

All data generated or analysed during this study are included in this published article and its supplementary information files (Supplemental Materials & Methods**;** Tables S1–S4 **;** Figures S1–S8 **;** Dataset S1–S5). The raw metabolomics data have been deposited to the ProteomeXchange Consortium via the PRIDE partner repository with the dataset identifier PXD061416&PXD061418, and 16S rRNA gene sequencing data have been deposited in the NCBI Sequence Read Archive (SRA) with the Bioproject ID PRJNA1221119.
