# Supplementary material for: Multi‐omics analysis revealed biomarkers for coronary atherosclerosis: Occurrence and development
Source: Clin Transl Med. 2025 Aug 21;15(8):e70451. doi: 10.1002/ctm2.70451 (PMC12371206; doi:10.1002/ctm2.70451)
Supplement: Supplementary file 6 — Supporting Information [file CTM2-15-e70451-s003.docx]

**Multi-omics analysis revealed biomarkers for coronary atherosclerosis****: occurrence and development**

Mengxue He^1^[[1]](#footnote-1)^#^, Dongxue Wang^2#^, Yong-Jiang Xu^1^, Jiachen Shi^1^, Aiyang Liu^1^, Xiaoxi Zhao^2^, Yunlai Gao^2^, Yuan He^1^, Yu Zhang^1^, Ru-Xing Wang^2*^, Yuanfa Liu^1^[[2]](#footnote-2)^*^

^1^ State Key Laboratory of Food Science and Technology, School of Food Science and Technology, National Engineering Research Center for Functional Food, National Engineering Laboratory for Cereal Fermentation Technology, Collaborative Innovation Center of Food Safety and Quality Control in Jiangsu Province, Jiangnan University, 214122 Wuxi, China.

^2^ Department of Cardiology, Wuxi People's Hospital Affiliated to Nanjing Medical University, 214023 Wuxi, China.

**2. SUPPLEMENTARY METHODS**

**2.1. Study design and population**

Coronary angiography (CAG) was performed in all patients after admission. The severity of coronary atherosclerosis (CA) was evaluated using the Gensini score (GS).^1^ The GS for grading lumen narrowing was: 1 for a 1% to 25% reduction in lumen diameter, 2 for a 26% to 50% reduction, 4 for a 51% to 75% reduction, 8 for a 76% to 90% reduction, 16 for a 91% to 99% reduction, and 32 for total occlusion. This score was multiplied by a factor that accounted for lesion position within the coronary arterial tree as follows: 5 for a left main lesion, 2.5 for a proximal left anterior descending and left circumflex lesion, and 1 for a proximal right coronary artery lesion. The severity of the disease was expressed as the sum of the scores of the individual lesions.

**2.4. 16S rRNA gene sequencing analysis**

Raw reads were filtered to remove adaptors and low-quality and ambiguous bases, and then paired-end reads were added to tags by the Fast Length Adjustment of Short reads program (FLASH, v1.2.11) to get the tags.^2^ The tags were clustered into OTUs with a cutoff value of 97% using UPARSE software (v7 .0.1090)^3^ and chimera sequences were compared with the Gold database using UCHIME (v4.2.40)^4^ to detect. Then, OTU representative sequences were taxonomically classified using Ribosomal Database Project (RDP) Classifier v.2.2 with a minimum confidence threshold of 0.6 and trained on the Greengenes database v201305 by QIIME v1.8.0.^5^ The USEARCH_global ^6^ was used to compare all Tags back to OTU to get the OTU abundance statistics table of each sample. Microbial diversity was examined by bioinformatics analysis (<http://www.microbiomeanalyst.ca/>; <http://huttenhower.sph.harvard.edu/galaxy>).

**2.5. Untargeted metabolomics analysis**

Sample preparation: During metabolite extraction of each sample, equal amounts of Fmoc-Gly were added to all samples as the internal standard. Plasma protein was precipitated by adding four volumes of ice-cold methanol (with) at room temperature and vortex mixing for 30 s. After centrifugation (12,000 g, 10 min, 4 ℃), the supernatants were collected for UPLC/QTOF-MS analysis. Fecal samples were dried under a low-temperature vacuum and ground into small particles. Approximately 3 mg of sample was added to 1.5 mL Eppendorf tubes with 200 μL of ice-cold MeOH: water (50:50, v/v) and Fmoc-Gly as an internal standard. The samples were sonicated in an ice bath for 30 min and then centrifuged at 12,000 g for 10 min. An 80 μL aliquot of the supernatant was transferred and dried in a vacuum concentrator. After drying, the sample was redissolved in 100 μL of ice-cold MeOH: water (50:50, v/v), and the above steps were repeated. Finally, an 80 μL aliquot of the supernatant was used for instrumental analysis.

Quality control (QC) samples were prepared by mixing equal volume from each sample to ensure consistency in retention time and signal intensity during the analytical process. In order to maintain the rigor of the analysis, QC samples were analyzed after every 15 samples throughout the mass spectrometry process. The plasma or fecal samples were randomly sequenced for analysis to mitigate any potential sequential bias, and samples were classified as blind, thus ensuring unbiased results.

HPLC-MS experiment parameters: Chromatographic analysis was performed on UPLC (AB SCIEX ExionLC) and TOF-MS (AB SCIEX, Triple TOF 5600^+^). The plasma samples were performed on a Kinetex C18 column (100 mm × 2.1 mm, 2.6 μm, Phenomenex), and a BEH amide column (100 mm × 2.1 mm, 1.7 μm, Waters). The feces samples were analyzed on a Kinetex C18 column (100 mm × 2.1 mm, 2.6 μm, Phenomenex), and a BEH Z-Hilic (100 mm×2.1 mm, 1.7μm, Thermo Scientific). During C18 column separation, the ingredients of the mobile phase were (A) water and (B) acetonitrile each containing 0.1% formic acid. The elution program was as follows: 0–1 min, 5% B; 1–11 min, 5–95% B; 11–13 min, 95% B; 13–14 min, 95–5% B; 14–15 min, 5% B. During BEH column separation, the mobile phase was composed of solvents A (10 mM ammonium formate in H_2_O) and B (10 mM ammonium formate in 90% acetonitrile/10% H2O). The elution program for plasma samples was as follows: 0–1 min, 100% B; 1–11 min, 100% B to 70% B; 11–12.5 min, 70% B; 12.5–13 min, 70% B to 100% B; 13–15 min, washing with 100% B. The elution program for fecal samples was as follows: 0–3 min, 95% B; 3–11 min, 95% B to 60% B; 11–12.5 min, 60% B to 95% B; 13–15 min, washing with 95% B. Data was collected in centroid mode from m/z 100 to m/z 1000 for fecal samples, and from m/z 50 to m/z 1000 for plasma samples.

Data processing and statistical analysis: The metabolic findings were gathered as *.wiff files, which MSConvert then transformed into *.mzXML files. Using MZmine 2.53 and Microsoft Excel 2020, all MS data was compiled, aligned, normalized, and peak tables were produced. The peak table was then uploaded to MetDNA for metabolite identification.^7^ The matrix was further reduced by removing peaks with missing values in more than 80% samples and those with isotope ions from each group to obtain consistent variables. By quantifying the internal standard compounds within each sample, ensure that the bias and coefficient of variation (CV) are within 15%, thus ensuring the stability of the analytical instrument and the reliability of the obtained data. The signal intensity of the analyte was compared with that of the standard to quantify the analyte. Subsequently, the relative quantitative analysis of metabolic substances was conducted based on the determination of the internal standard content. The processed data list was then imported to SIMCA-P (version 14.1) for orthogonal partial least-squares-discriminant analysis (OPLS-DA). The nonparametric univariate method was used to analyze metabolites that differed in abundance between the different subgroups to ensure that the peak of each metabolite was reproducibly detected in the samples. The parameters R^2^X and Q^2^Y were stable and good for fitness and prediction. The difference metabolites were screened according to the fold change (FC) against control samples. Using R to draw a heatmap to visualize the relative content of different metabolites in each group. MetaboAnalyst (https://www.metaboanalyst.ca) was used for the identification of metabolic pathways.

**2.6. Targeted metabolomics analysis of biomarkers**

A targeted analysis was used to quantify the identified biomarkers by multiple reaction monitoring (MRM). The potential markers were quantified with MRM mode by QTRAP 5500 (SCIEX, USA) coupled with UPLC (Waters, USA) using the same LC condition, as described in discovery stage. The parent and product ion pairs with high sensitivity and selectivity were selected as the final MRM ion pairs for quantitative analysis. To further improve the ionization efficiency and achieve maximum sensitivity, we further optimized the DP and CE of each ion pair. The MRM parameters including ion pair, DP, CE, and RT are listed in Table S4. Compounds confirmed by reference standards under the same analytical conditions, including matched retention time (RT), primary (MS1), and secondary (MS2).

According to the detected concentrations of the corresponding metabolites in plasma, the standard solution of the corresponding concentration gradient was prepared, and the standard curve was drawn by regression analysis with the concentration of the standard substance as the horizontal coordinate and the peak area of the standard substance as the vertical coordinate. Table S4 shows the linear correlation equations and R^2^ of 25 metabolites.

**3. SUPPLEMENTARY RESULTS**

**Associations between gut microbiota, plasma metabolome, and clinical feature**

We assessed the associations between the plasma level of significantly different clinical parameters including GFR, HDL-C, ApoB, TC, LDL-C, creatinine, UA, FBG, ALP, LPA, and TLC with the gut microbiota (Figure S4A, Supplementary Dataset S3). We found that only TLC was positively correlated with *Haemophilus****.*** As for the plasma level of metabolites (34 plasma metabolites significantly differed in all AS stages compared with Ctr), we found that except for LPA, these clinical variables were negatively and/or positively correlated with at least one of the plasma metabolites. Specifically, there was a positive correlation between the plasma level of LPC 18:2 and HDL-C, ApoB, TC, and LDL-C, and a negative correlation between these variables and the plasma level n2-gamma-glutamylglutamine. The plasma level of 2-aminoadipic acid (2AA), *L*-glutamic acid, *L*-glutamate, and *L*-pyroglutamic acid (PGA) showed a positive correlation with UA and FBG, and a negative related to HDL-C (Figure S4C, Supplementary Dataset S3)***.*** Then we evaluated the relationships between the dramatically changed gut microbe and the plasma levels of metabolites. We found some genera of *Bacteroides*, *Bifidobacterium*, and *Haemophilus* were positively associated with LPE 18:2. However, there was a negative correlation between the genus of *Streptococcus* with LPC 18:2, 3-(1-pyrazolyl)-alanine, 3-methyluridine, N2-gamma-glutamylglutamine, and ADMA (Figure S4B, Supplementary Dataset S3). Interestingly, the abundance of the *Lactobacillus* was positively correlated with TMAO, PAGln, and 2AA. In addition, LPC 18:2, and LPE 18:2 plasma level was positively correlated with the abundances of *Haemophilus.* Azelaic acid (AA) and LPE 18:2 plasma levels were positively correlated with the abundance of *Bifidobacterium*.

**Table S1** Sensitivity, specificity and area under the curve (AUC) of predication model.

|  | Metabolites | Sensitivity | Specificity | AUC | 95%CI |
| --- | --- | --- | --- | --- | --- |
| Ctr vs AS0 | Cholesteryl sulfate | 0.900 | 0.929 | 0.936 | 0.796-0.991 |
|  | Azelaic acid | 0.750 | 0.857 | 0.879 | 0.721-0.965 |
|  | Tryptophan | 0.900 | 0.786 | 0.843 | 0.677-0.944 |
|  | Arabinofuranosyluracil | 0.900 | 0.786 | 0.836 | 0.669-0.940 |
|  | TMAO | 0.900 | 0.786 | 0.814 | 0.644-0.926 |
|  | Combination | 0.900 | 1.000 | 0.979 | 0.859-1.000 |
| Ctr vs AS1 | ADMA | 1.000 | 0.714 | 0.904 | 0.780-0.971 |
|  | LPC18:2 | 0.781 | 0.927 | 0.891 | 0.763-0.963 |
|  | Tartaric acid | 0.656 | 0.929 | 0.810 | 0.668-0.911 |
|  | L-Citrulline | 0.719 | 0.643 | 0.712 | 0.560-0.836 |
|  | L-Proline | 0.875 | 0.571 | 0.663 | 0.509-0.796 |
|  | Combination | 0.969 | 1.000 | 0.998 | 0.919-1.000 |
|  | TMAO | 0.889 | 1.000 | 0.937 | 0.814-0.989 |
|  | ADMA | 0.889 | 0.714 | 0.857 | 0.712-0.947 |
| Ctr vs AS2 | Purine | 0.852 | 1.000 | 0.852 | 0.706-0.943 |
|  | Sorbitol | 0.667 | 0.929 | 0.799 | 0.645-0.908 |
|  | 2-Aminoadipic acid | 0.512 | 1.000 | 0.799 | 0.645-0.908 |
|  | Combination | 1.000 | 1.000 | 1.000 | 0.914-1.000 |
| AS0 vs AS1 | Tryptophan | 0.852 | 0.950 | 0.881 | 0.754-0.957 |
|  | L-Pyroglutamic acid | 0.889 | 0.700 | 0.863 | 0.731-0.946 |
|  | Inosine | 0.593 | 0.950 | 0.804 | 0.662-0.905 |
|  | Myristic acid | 0.889 | 0.700 | 0.787 | 0.643-0.893 |
|  | L-Threonate | 0.667 | 0.850 | 0.722 | 0.572- 0.843 |
|  | Combination | 0.938 | 0.800 | 0.933 | 0.828-0.984 |
| AS1 vs AS2 | TMAO | 0.889 | 1.000 | 0.896 | 0.789-0.960 |
|  | 2-Oxobutyric acid | 0.852 | 0.656 | 0.807 | 0.683-0.898 |
|  | 4-Pyridoxic acid | 0.444 | 0.906 | 0.727 | 0.595-0.835 |
|  | Theophylline | 0.889 | 0.563 | 0.698 | 0.564-0.811 |
|  | 3-Hydroxybutyric acid | 0.778 | 0.563 | 0.659 | 0.524-0.777 |
|  | Combination | 0.889 | 0.969 | 0.965 | 0.882-0.996 |
| AS0 vs AS2 | TMAO | 0.852 | 0.950 | 0.881 | 0.754-0.957 |
|  | L-Tyrosine | 0.889 | 0.700 | 0.863 | 0.731-0.946 |
|  | Beta-Indoleacetic acid | 0.593 | 0.950 | 0.804 | 0.662-0.905 |
|  | Theanine | 0.889 | 0.700 | 0.787 | 0.643-0.893 |
|  | Hypoxanthine | 0.667 | 0.850 | 0.722 | 0.572-0.843 |
|  | Combination | 1.000 | 1.000 | 1.000 | 0.925-1.000 |

**Table S2** Baseline characteristics in the validation cohorts.

| Characteristics | Mean ± SD (n = 104) |
| --- | --- |
| Male, n % | 65.38% |
| BMI, kg/m^2^ | 24.32±2.99 |
| Age, year | 60.23±11.95 |
| Systolic pressure, mm Hg | 137.9±18.56 |
| Diastolic pressure, mm Hg | 76.42±12.14 |
| Total cholesterol, mmol/L | 4.22±1.34 |
| Triglyceride, mmol/L | 1.67±0.97 |
| HDL-cholesterol, mmol/L | 1.09±0.26 |
| LDL-cholesterol, mmol/L | 2.35±1.03 |
| Apolipoprotein A, mmol/L | 1.36±0.25 |
| Apolipoprotein B, mmol/L | 0.74±0.25 |
| Fasting blood-glucose, mmol/L | 5.05±0.78 |
| Creatinine, μmol/L | 73.39±16.84 |
| Blood urea nitrogen, mmol/L | 5.62±1.46 |
| Uric acid, μmol/L | 360.75±81.15 |
| Glomerular filtration rate, mL/min | 91.36±15.33 |
| Alanine transaminase, U/L | 23.74±15.02 |
| Aspartate transaminase, U/L | 24.97±12.92 |
| Alkaline phosphatase, U/L | 74.79±23.17 |
| Gamma-glutamyl transferase, U/L | 26.77±14.18 |
| Total protein, g/L | 66.11±5.22 |
| Albumin/Globulin | 1.53±0.25 |
| Total bilirubin, μmol/L | 14.93±6.41 |
| hs-CRP, mg/L | 2.81±2.68 |

**Table S3** The results of logistic regression adjustment for confounding factors.

|  |  | Unadjusted results | | |  | Adjusted results* | | |
| --- | --- | --- | --- | --- | --- | --- | --- | --- |
|  | Metabolites | *P* value | OR | 95%CI |  | *P* value | OR | 95%CI |
| Ctr vs AS0 | Cholesteryl sulfate | 0.012 | 0.240 | 0.079-0.730 |  | 0.017 | 0.160 | 0.039-0.664 |
|  | Azelaic acid | 0.016 | 0.003 | 0.001-0.023 |  | 0.021 | 0.001 | 0.001-0.003 |
|  | Tryptophan | 0.008 | 0.830 | 0.724-0.953 |  | 0.019 | 0.688 | 0.503-0.941 |
|  | Arabinofuranosyluracil | 0.037 | 0.763 | 0.592-0.984 |  | 0.054 | 0.776 | 0.600-1.004 |
|  | TMAO | 0.005 | 1.641 | 1.164-2.315 |  | 0.023 | 3.370 | 1.186-09.578 |
| Ctr vs AS1 | ADMA | 0.006 | 2.456 | 1.253-3.710 |  | 0.047 | 1.810 | 1.009-3.246 |
|  | LPC18:2 | 0.001 | 0.954 | 0.927-0.982 |  | 0.022 | 0.920 | 0.856-0.988 |
|  | Tartaric acid | 0.011 | 0.006 | 0.001-0.316 |  | 0.016 | 0.002 | 0.001-0.166 |
|  | L-Citrulline | 0.021 | 0.867 | 0.768-0.978 |  | 0.040 | 0.840 | 0.712-0.992 |
|  | L-Proline | 0.034 | 0.976 | 0.954-0.998 |  | 0.121 | 0.978 | 0.950-1.007 |
|  | TMAO | 0.001 | 1.338 | 1.127-1.587 |  | 0.105 | 1.916 | 0.872-4.207 |
|  | ADMA | 0.013 | 4.249 | 1.356-13.316 |  | 0.025 | 6.691 | 1.274-35.147 |
| Ctr vs AS2 | Purine | 0.003 | 0.003 | 0.001-0.140 |  | 0.026 | 0.008 | 0.005-0.235 |
|  | Sorbitol | 0.010 | 6.020 | 3.563-8.356 |  | 0.013 | 9.329 | 5.329-11.764 |
|  | 2-Aminoadipic acid | 0.007 | 1.487 | 1.113-1.985 |  | 0.016 | 1.478 | 1.074-2.035 |
|  |  | Unadjusted results | | |  | Adjusted results* | | |
|  | Metabolites | *P* value | OR | 95%CI |  | Adjusted *P* value **^*^** | OR | 95%CI |
| AS0 vs AS1 | Tryptophan | 0.003 | 1.118 | 1.038-1.204 |  | 0.004 | 1.133 | 1.042-1.233 |
|  | L-Pyroglutamic acid | 0.003 | 0.827 | 0.729-0.938 |  | 0.004 | 0.800 | 0.693-0.922 |
|  | Inosine | 0.002 | 3.632 | 1.596-5.888 |  | 0.003 | 5.274 | 2.276-7.686 |
|  | Myristic acid | 0.011 | 0.769 | 0.628-0.942 |  | 0.018 | 0.776 | 0.628-0.958 |
|  | L-Threonate | 0.015 | 1.185 | 1.033-1.359 |  | 0.019 | 1.217 | 1.033-1.433 |
| AS1 vs AS2 | TMAO | <0.001 | 1.456 | 1.195-1.773 |  | <0.001 | 1.500 | 1.204-1.869 |
|  | 2-Oxobutyric acid | 0.001 | 4.896 | 1.679-7.926 |  | 0.002 | 6.112 | 2.161-9.250 |
|  | 4-Pyridoxic acid | 0.004 | 0.767 | 0.641-0.918 |  | 0.010 | 0.765 | 0.624-0.937 |
|  | Theophylline | 0.048 | 0.196 | 0.039-0.985 |  | 0.010 | 0.030 | 0.002-0.435 |
|  | 3-Hydroxybutyric acid | 0.080 | 0.939 | 0.875-1.007 |  | 0.101 | 0.942 | 0.876-1.012 |
| AS0 vs AS2 | TMAO | 0.001 | 1.311 | 1.118-1.537 |  | 0.002 | 1.369 | 1.123-1.668 |
|  | L-Tyrosine | 0.001 | 1.460 | 1.172-1.818 |  | 0.007 | 2.194 | 1.239-3.886 |
|  | Beta-Indoleacetic acid | 0.005 | 4.130 | 2.523-6.155 |  | 0.007 | 6.185 | 3.520-9.707 |
|  | Theanine | 0.048 | 1.756 | 0.956-3.239 |  | 0.060 | 2.298 | 0.966-5.469 |
|  | Hypoxanthine | 0.009 | 2.870 | 1.308-4.295 |  | 0.011 | 3.516 | 1.462-5.953 |

^*^ Adjusted results were adjusted for confounding factors (age, sex, body mass index, systolic pressure, diastolic pressure, total cholesterol, triglyceride, lipoprotein A, HDL-cholesterol, LDL—cholesterol, apolipoprotein A, apolipoprotein B, fasting blood-glucose, blood urea nitrogen, uric acid, glomerular filtration rate, alanine transaminase, aspartate transaminase, alkaline phosphatase, gamma-glutamyl transferase, total protein, albumin/globulin, total bilirubin, total leukocyte count, fibrinogen and D-dimer).

**Table S4** Optimized multiple reaction monitoring parameters and calibration curves for the quantitative analysis of diagnostic biomarkers.

| Component Name | Precursor Mass  (m/z) | Fragment Mass  (m/z) | DP  (eV) | CE  (eV) | RT  (min) | Standard curve | **R^2^** |
| --- | --- | --- | --- | --- | --- | --- | --- |
| LPC 18:2 | 520.2 | 184.1 | +70 | +32 | 5.54 | y = 7256163 x + 1351472 | 0.9999 |
| Trimethylamine-N-oxide | 76.1 | 58.1 | +75 | +26 | 5.71 | y = 813115976 x + 30503 | 0.9999 |
| Purine | 120.9 | 94.1 | +80 | +24 | 2.61 | y = 33744787 x + 25778 | 0.9939 |
| L-Pyroglutamic acid | 130.1 | 84.1 | +83 | +18 | 2.14 | y = 48360119 x + 917624 | 0.9965 |
| ADMA | 203.1 | 70.1 | +80 | +33 | 11.23 | y = 15976442 x + 44060 | 0.9997 |
| Hypoxanthine | 137.0 | 110.1 | +105 | +30 | 4.32 | y = 59586920 x - 21821 | 0.9950 |
| L-Tyrosine | 182.1 | 165.1 | +65 | +18 | 7.55 | y = 4469428 x + 56141 | 0.9968 |
| Sorbitol | 183 | 129 | +80 | +15 | 6.59 | y = 564394x - 88.059 | 0.9908 |
| Beta-Indoleacetic acid | 175.9 | 129.9 | +80 | +15 | 1.01 | y = 4200358 x - 30298 | 0.9990 |
| Cholesteryl sulfate | 465.3 | 96.5 | -80 | -110 | 1.02 | y = 3302945 x - 91603 | 0.9947 |
| Azelaic acid | 187.1 | 125.1 | -90 | -25 | 1.05 | y = 3552218 x + 83702 | 0.9988 |
| Myristic acid | 227.5 | 85.2 | -80 | -50 | 2.13 | y = 5711 x + 33682 | 0.9800 |
| Arabinofuranosyluracil | 243.1 | 110.1 | -80 | -25 | 4.13 | y = 103761 x + 3839 | 0.9951 |
| 3-Hydroxybutyric acid | 103.1 | 41.3 | -80 | -35 | 1.23 | y = 964301 x + 22055 | 0.9950 |
| Theanine | 173.3 | 84.3 | -80 | -23 | 7.67 | y = 2513659x + 952 | 0.9958 |
| Tryptophan | 203.3 | 116.3 | -80 | -25 | 6.88 | y = 4684619 x + 16770 | 0.9985 |
| Inosine | 267.1 | 135.1 | -80 | -50 | 5.25 | y = 61614432 x - 2822 | 0.9987 |
| Tartaric acid | 149.1 | 73.3 | -60 | -22 | 3.49 | y = 32654 x + 7196 | 0.9913 |
| L-Threonate | 135.1 | 117.1 | -60 | -15 | 4.26 | y = 4469428 x + 56141 | 0.9999 |
| 2-Oxobutyric acid | 101.3 | 57.5 | -70 | -32 | 1.32 | y = 6128175 x - 1916 | 0.9987 |
| 4-Pyridoxic acid | 184.1 | 166.0 | +80 | +20 | 8.14 | y = 32654 x + 7196 | 0.9913 |
| L-Citrulline | 175.9 | 158.9 | +60 | +14 | 9.49 | y = 23524692 x - 27651 | 0.9998 |
| L-Proline | 116.1 | 70.1 | +100 | +21 | 7.17 | y = 31958209 x + 17150734 | 0.9987 |
| Theophylline | 181.5 | 123.7 | +70 | +25 | 1.45 | y = 2779812 x + 1578 | 0.9913 |
| 2-Aminoadipic acid | 162.1 | 116.1 | +80 | +32 | 4.92 | y = 5187940 x + 2193 | 0.9994 |

DP: declustering potential; CE: collision energy; RT: retention time; R^2^: coefficient of determination

**
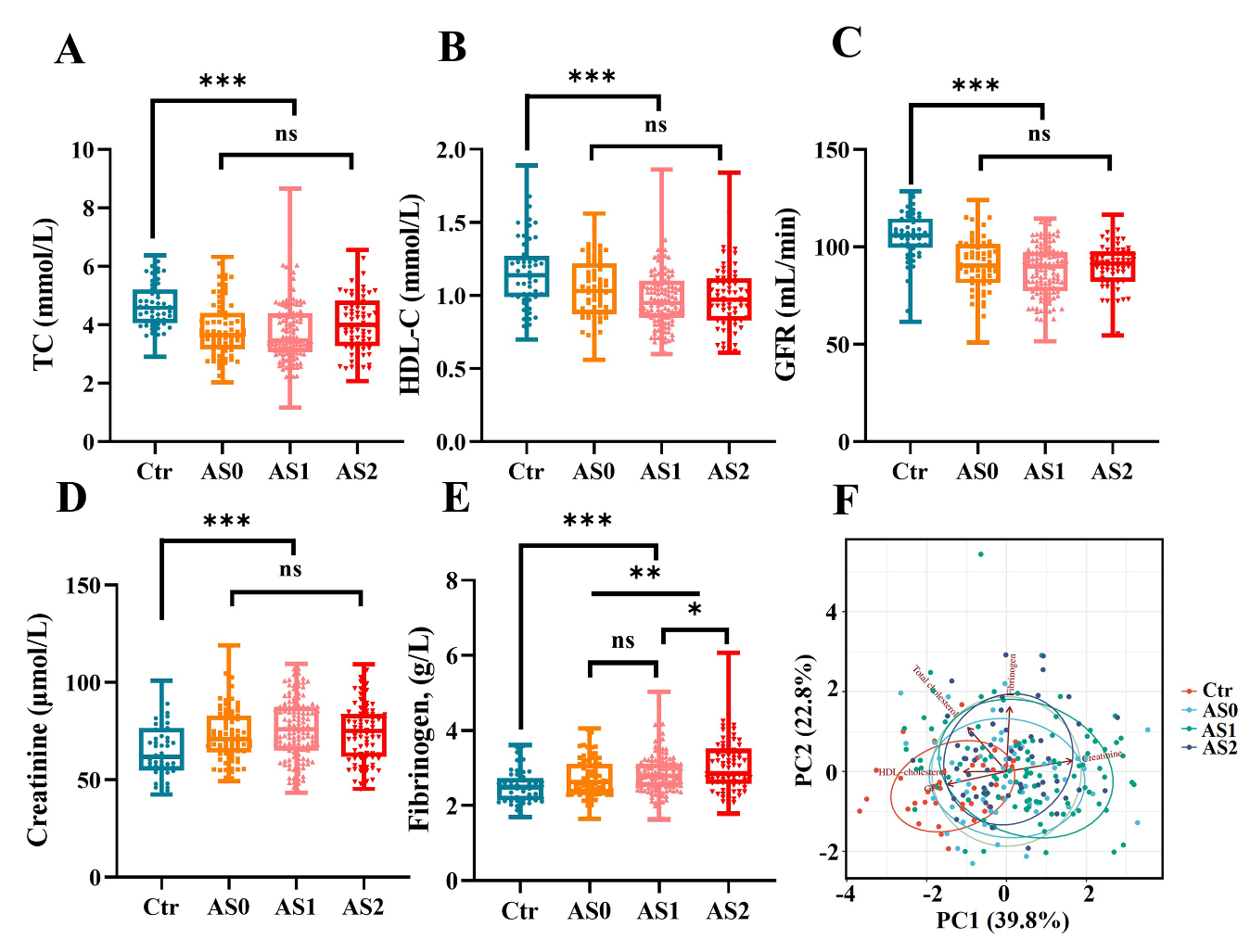
**

**FIGURE S1** Significantly different clinical parameters in both training and testing population. (A-E) Significantly different clinical parameters are presented in subjects with Ctr, AS0, AS1, and AS2. Student’s t-test was used for statistical analysis; (F) Principal components analysis (PCA) of all subjects based on five significantly different clinical variables.


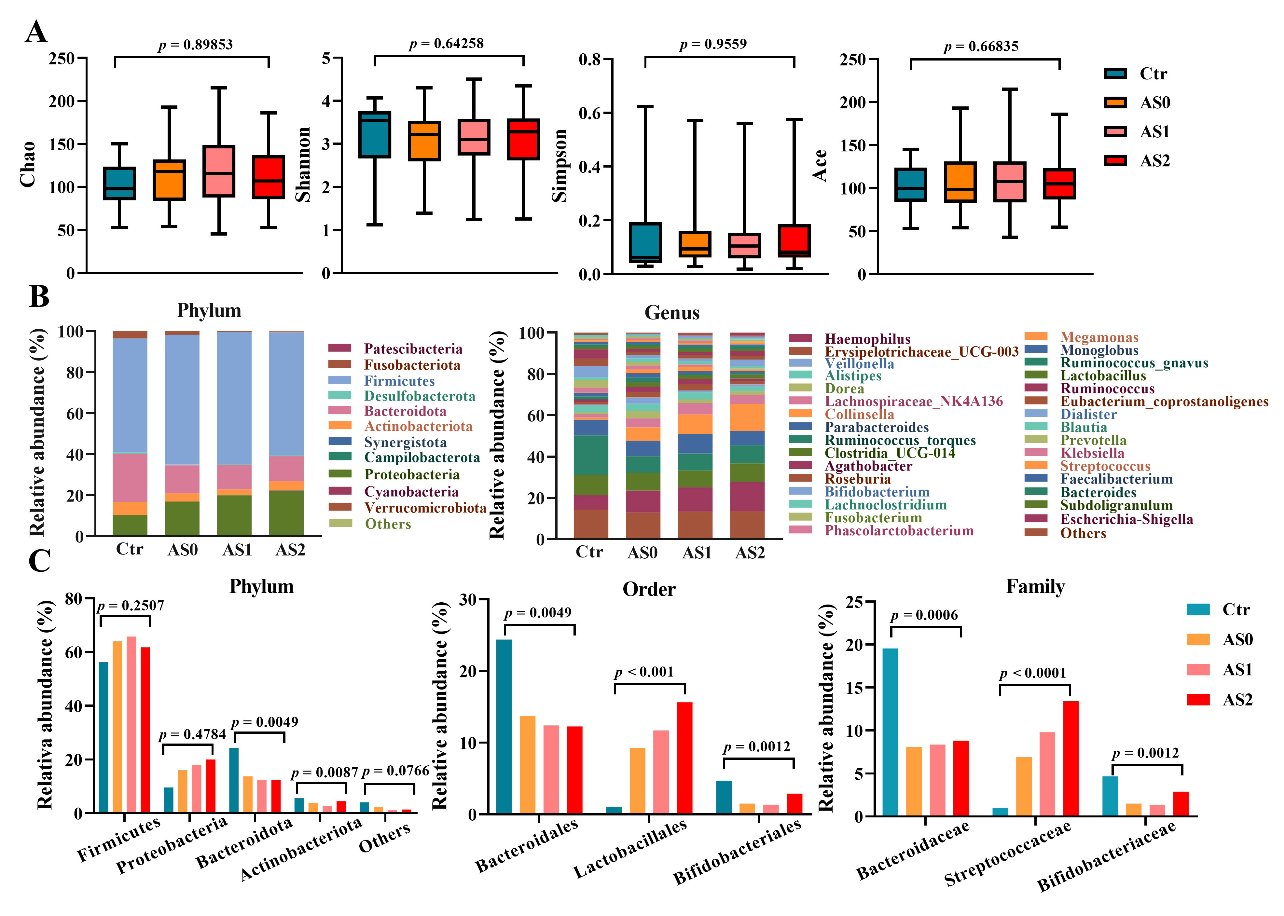


**FIGURE S2** Comparison of the microbial community and fecal metabolites. (A)Alpha diversity; (B) Changes and differences in microbiota on phylum and genus level; (C) The abundant in phyla, orders, and families, of the gut microbiota between coronary atherosclerosis and healthy control.


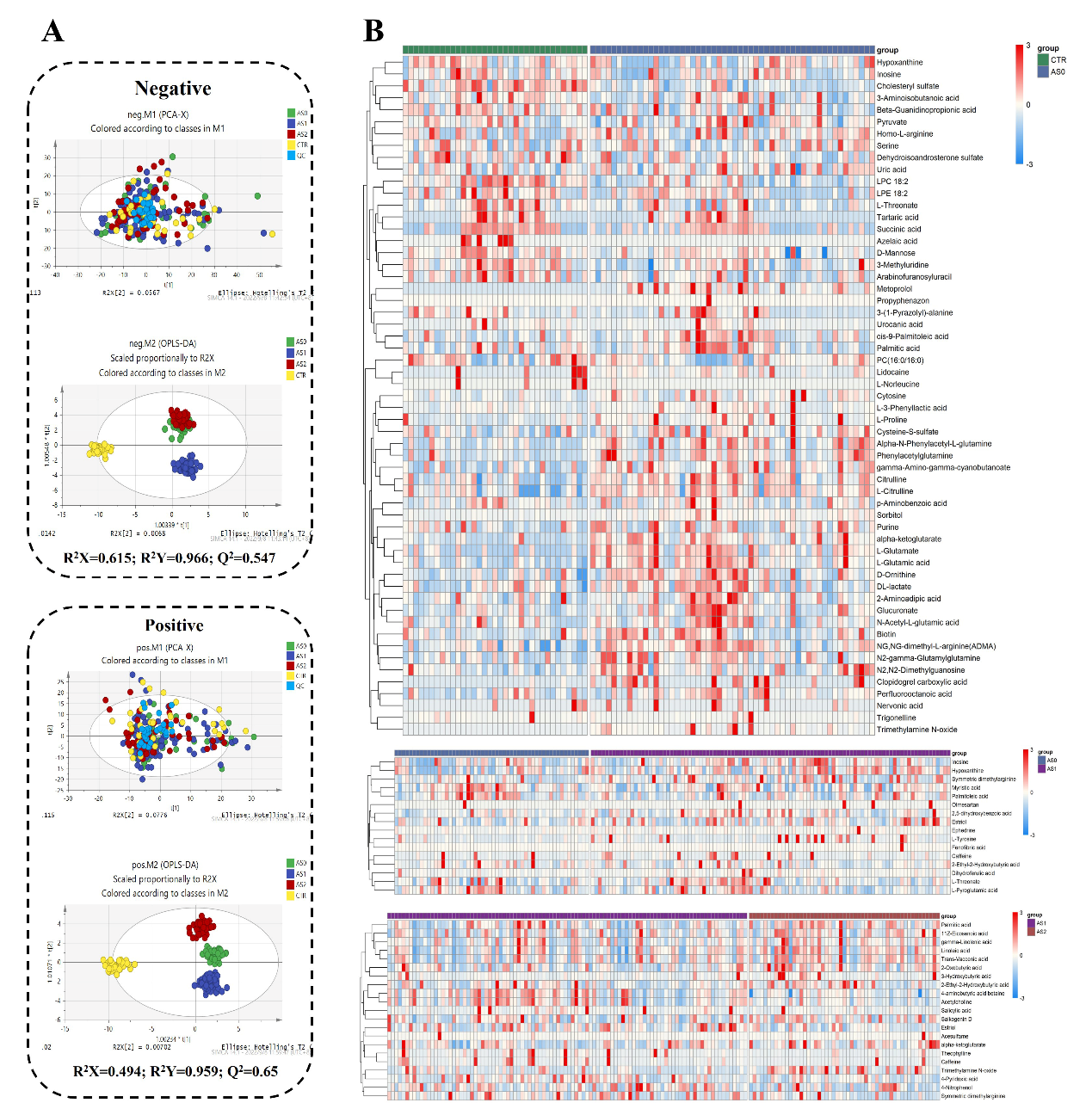


**FIGURE S3** Metabolite profile of plasma sample in different groups. **(**A) principal component analysis (PCA) and orthogonal partial least-squares-discriminant analysis (OPLS-DA) score plots; (B) A heat map of the a significantly changed metabolites between Ctr vs AS0, AS0 vs AS1, and AS1 vs AS2 (*p* <0.05).


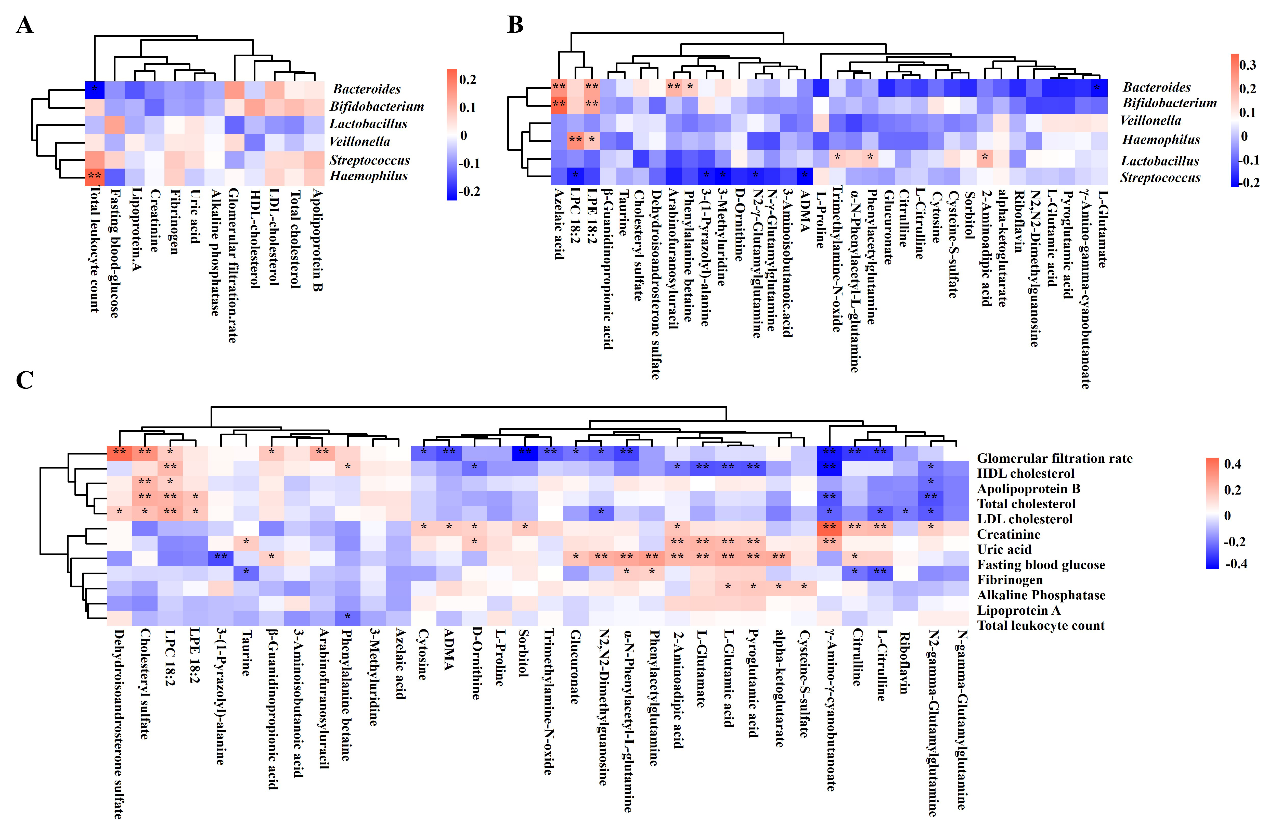


**FIGURE S4.** Correlation heatmap of the altered gut microbiota, clinical feature and plasma metabolites. (A) Gut microbiota and clinical feature; (B) Gut microbiota and plasma metabolites. C: Clinical feature and plasma metabolites. Blue squares indicate significant negative correlations (r < -0.5, *p* < 0.05), white squares indicate nonsignificant correlations (*p* > 0.05), and red squares indicate significant positive correlations (r > 0.5, *p* < 0.05) *: *p* < 0.05, **: *p* < 0.01.


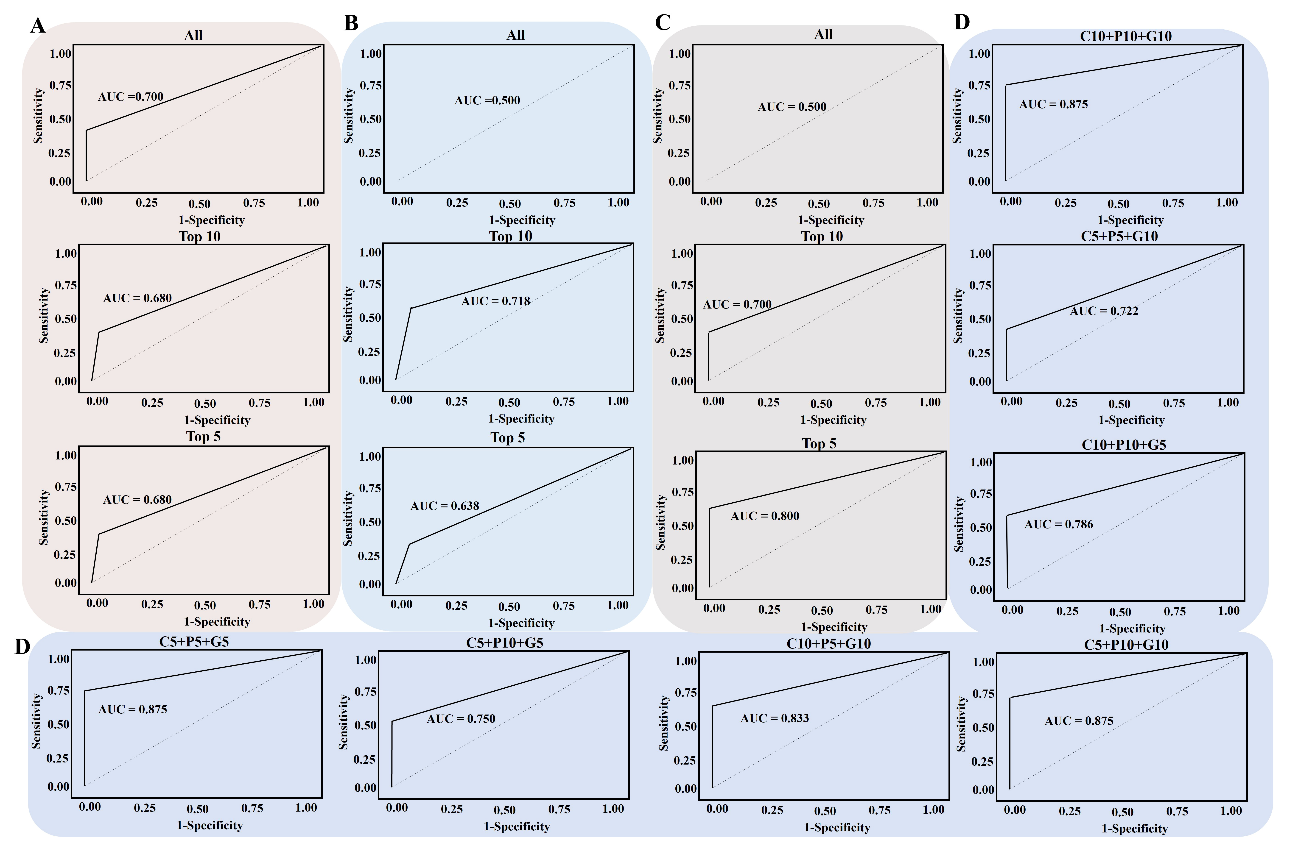


**FIGURE S5** Receiver operating characteristic (ROC) analysis to predict Ctr from AS group using single omics. (A) Clinical parameters, (C); (B) Gut microbiota, (G); (C) Plasma metabolites, (P); (D) Multi-omics combination of top features from each omics.


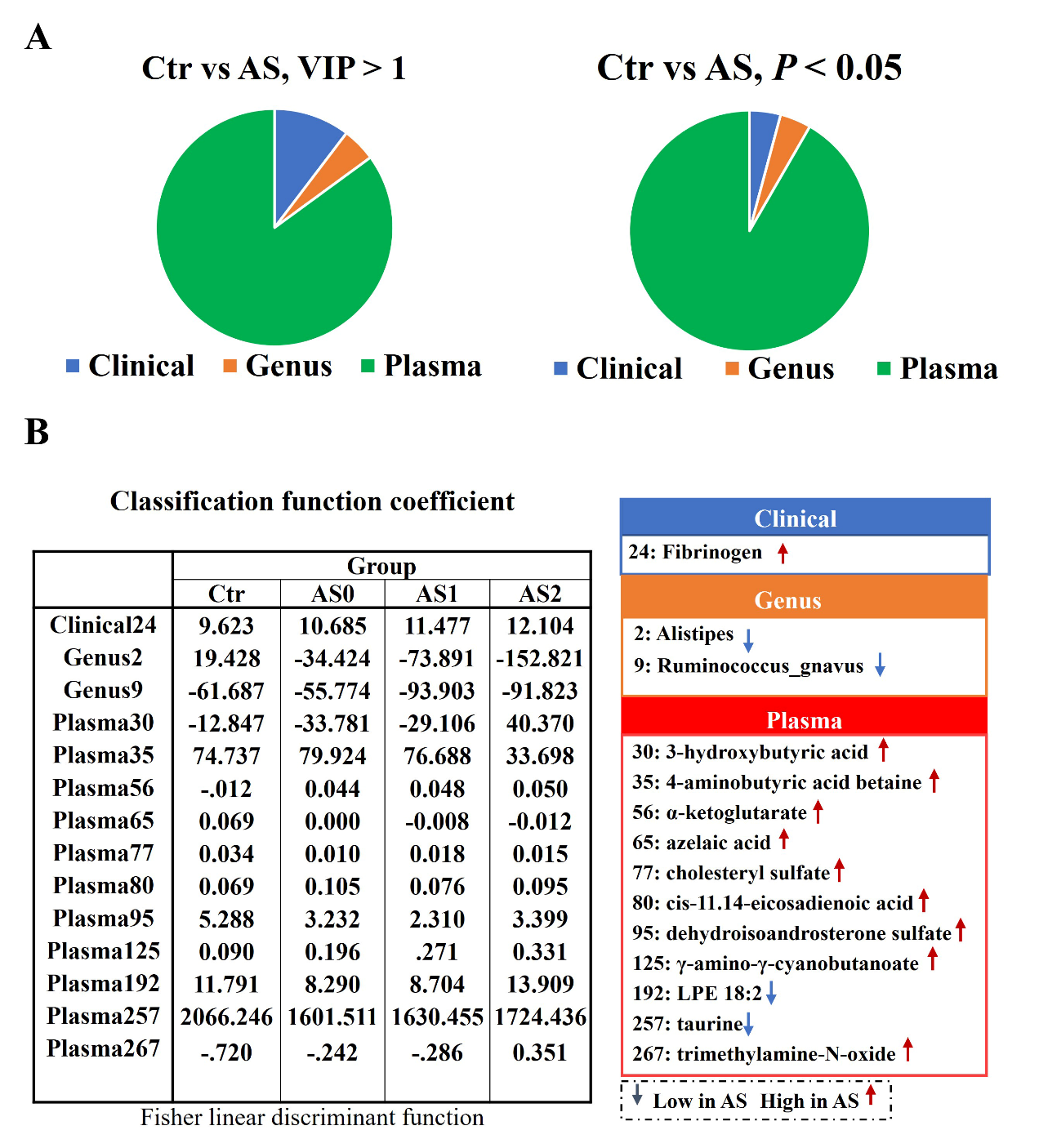


**FIGURE S6** Single/multiomics data identify biomarkers for distinguishing the severity of coronary atherosclerosis. (A) The results of orthogonal partial least-squares-discriminant analysis (OPLS-DA) model (VIP >1) and ANOVA (*p* < 0.05) compared Ctr vs AS0 vs AS1 vs AS2; (B) Fisher discriminant analysis results about the calculation of subgroup.


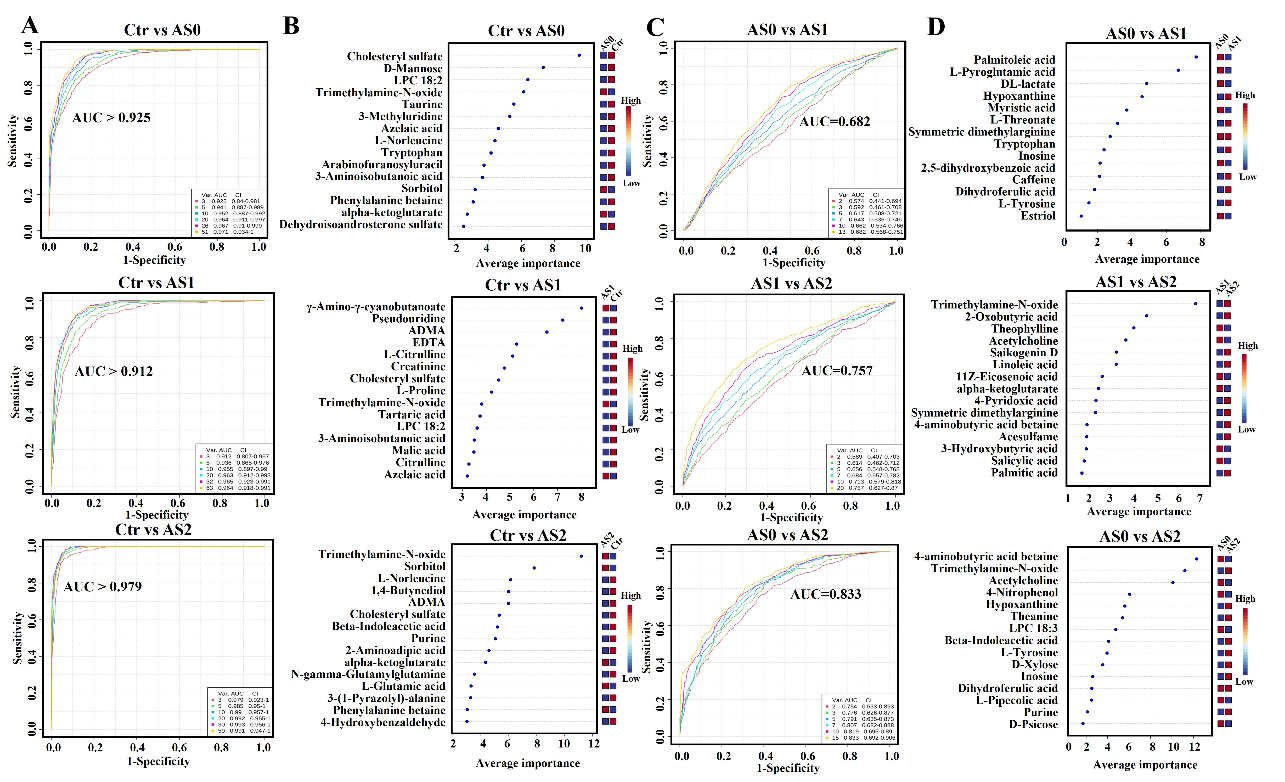


**FIGURE S7** Plasma metabolites markers for pairwise discriminations of Ctr, AS0 and AS1 groups. **(**A) Receiver operating characteristic (ROC) analysis to discriminate Ctr from AS groups; (B) Top 15 features from the Ctr vs AS model with the highest accuracy; (C) ROC analysis to discriminate AS0 and AS1, AS1 and AS2, and AS0 and AS2; (D) Top 15 features from the AS degree model with the highest accuracy.


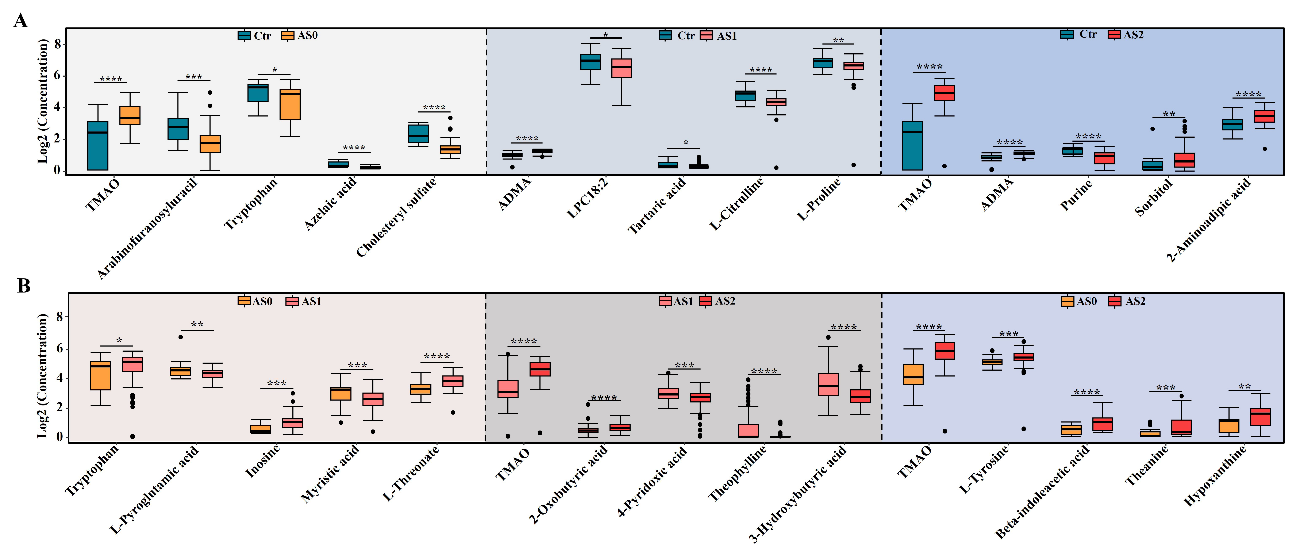


**FIGURE S8** Quantified analysis of biomarkers in validation cohort study. (A) Biomarker panels for predict the occurrence of coronary atherosclerosis; (B) Biomarker panels for predict the severity of coronary atherosclerosis.

**Reference**

1. Gensini GG. A more meaningful scoring system for determining the severity of coronary heart disease. *Am J Cardiol.* 1983;51(3):606.

2. Magoč T, Salzberg SL. FLASH: fast length adjustment of short reads to improve genome assemblies. *Bioinformatics.* 2011;27(21):2957-2963.

3. Edgar RC. UPARSE: highly accurate OTU sequences from microbial amplicon reads. *Nat Methods.* 2013;10(10):996-998.

4. Edgar RC, Haas BJ, Clemente JC, Quince C, Knight R. UCHIME improves sensitivity and speed of chimera detection. *Bioinformatics.* 2011;27(16):2194-2200.

5. Caporaso JG, Kuczynski J, Stombaugh J, et al. QIIME allows analysis of high-throughput community sequencing data. *Nat Methods.* 2010;7(5):335-336.

6. Edgar RC. Search and clustering orders of magnitude faster than BLAST. *Bioinformatics.* 2010;26(19):2460-2461.

7. Shen X, Wang R, Xiong X, et al. Metabolic reaction network-based recursive metabolite annotation for untargeted metabolomics. *Nat Commun.* 2019;10(1):1516.

1. # These authors contributed equally to this work. [↑](#footnote-ref-1)
2. * Corresponding author’E-mail: ruxingw@njmu.edu.cn (R. X Wang)

   * Corresponding author’E-mail: [yfliu@jiangnan.edu.cn](mailto:yfliu@jiangnan.edu.cn) (Y. F Liu) [↑](#footnote-ref-2)
